# PMS2 has both pro-mutagenic and anti-mutagenic effects on repeat instability in the Repeat Expansion Diseases

**DOI:** 10.1101/2024.08.13.607839

**Published:** 2024-08-13

**Authors:** Alexandra Walker, Diego Antonio Jimenez, Karen Usdin, Xiaonan Zhao

**Affiliations:** 1 Section on Gene Structure and Disease, Laboratory of Cell and Molecular Biology, National Institute of Diabetes and Digestive and Kidney Diseases, National Institutes of Health, Bethesda, MD 20892

**Keywords:** microsatellite instability, mismatch repair (MMR), MutLα, MutLγ, Huntington’s disease, Fragile X-related disorders

## Abstract

Genome Wide Association studies (GWAS) have implicated PMS2 as a modifier of somatic expansion in Huntington’s disease (HD), one of >45 known Repeat Expansion Diseases (REDs). PMS2 is a subunit of the MutLα complex, a major component of the mismatch repair (MMR) system, a repair pathway that is involved in the generation of expansions in many different REDs. However, while MLH3, a subunit of a second MutL complex, MutLγ, is required for all expansions, PMS2 has been shown to protect against expansion in some model systems but to drive expansion in others. To better understand PMS2’s behavior, we have compared the effect of the loss of PMS2 in different tissues of an HD mouse model (CAG/CTG repeats) and a mouse model for the Fragile X-related disorders (FXDs), disorders that result from a CGG/CCG repeat expansion. Mice heterozygous for *Pms2* show increased expansions in most expansion-prone tissues in both disease models. However, in *Pms2* null mice expansions of both repeats increased in some tissues but decreased in others. Thus, the previously reported differences in the effects of PMS2 in different model systems do not reflect fundamentally different roles played by PMS2 in different REDs, but rather the paradoxical effects of PMS2 in different cellular contexts. These findings have important implications not only for the mechanism of expansion and the development of therapeutic approaches to reduce the pathology generated by repeat expansion, but also for our understanding of normal MMR.

## Introduction

Repeat expansion, the increase in the number of repeats in a short tandem repeat (STR) in a disease-specific gene, is the cause of the Repeat Expansion Diseases (REDs), a group of >45 neurological or neurodevelopmental disorders^1^. Converging evidence from studies of genetic modifiers in patients with different REDs implicates components of the Mismatch Repair (MMR) pathway as modifiers of both repeat expansion and disease severity^2–6^. This has raised the possibility that targeting some of these factors may be useful therapeutically^7^, an appealing idea since these diseases currently have no effective treatment or cure. Some of these same factors have been implicated in expansion in different cell and mouse models of these disorders^8–12^. Taken together these findings suggest that different REDs may expand using the same or very similar mechanisms and that these cell and mouse models are suitable for understanding the expansion process in humans.

However, discordant effects have been seen in different diseases and disease models for some of the MMR factors, casting doubt on the idea that all REDs share a common mechanism or, at least, suggesting that there are important differences between diseases for some important genetic modifiers. For example, PMS2, a subunit of the MutLα complex, one of the two MutL complexes involved in lesion processing in mammalian MMR, has been shown to promote repeat expansion in some models but to protect against it in others. Genome wide association studies (GWAS) have shown that PMS2 is a modifier of age at onset and risk of somatic expansion in Huntington’s disease (HD)^13,14^, a CAG-repeat expansion disorder^15^. Somatic expansion in HD has been linked to modifier haplotypes that are associated with both earlier and later onset^5,14^. PMS2 has also been shown to protect against expansion in the brain^16,17^ and liver^16^ of HD mouse models as well as in the brain of a mouse model of Friedreich’s ataxia (FRDA)^18^, a GAA-repeat expansion disorder^19^. However, PMS2 was shown to be required for expansion in mouse embryonic stem cells (mESCs) from a mouse model of the Fragile X-related disorders (FXDs)^20^, which are caused by CGG-repeat expansion in the Fragile X Messenger Ribonucleoprotein 1 (*FMR1*) gene^21^. This requirement for PMS2 was similarly observed in induced pluripotent stem cells (iPSCs) derived from a patient with Glutaminase Deficiency (GLSD)^11^, a CAG-repeat expansion disorder^22^, where the absence of PMS2 resulted in loss of all expansions. Furthermore, in a mouse model of Myotonic Dystrophy Type 1 (DM1), a CTG-repeat expansion disorder^23^, loss of PMS2 results in the loss of ~50% of expansions in many organs^24^.

To understand these differences, we carried out a systematic comparison of the effect of the loss of PMS2 in different organs of two different REDs mouse models, a mouse model of HD and a mouse model of the FXDs, using animals matched for age and repeat number and having the same genetic background. We found that with a few notable exceptions, PMS2 has very similar effects on repeat expansion at both loci in these mouse models. However, these effects are paradoxical since PMS2 was found to promote expansion in some contexts and suppress it in others in a manner that depended on gene dosage. Thus, the differences previously observed with respect to PMS2’s effects in different model systems likely do not reflect different expansion mechanisms in different disease models, but rather the different roles that PMS2 plays in different cell contexts. Our findings provide insight into the expansion mechanism and are relevant for efforts to target components of the MMR pathway to reduce somatic expansion.

## Results

### PMS2 plays a dual role in somatic expansion in an FXD mouse model.

**Fig.1.**
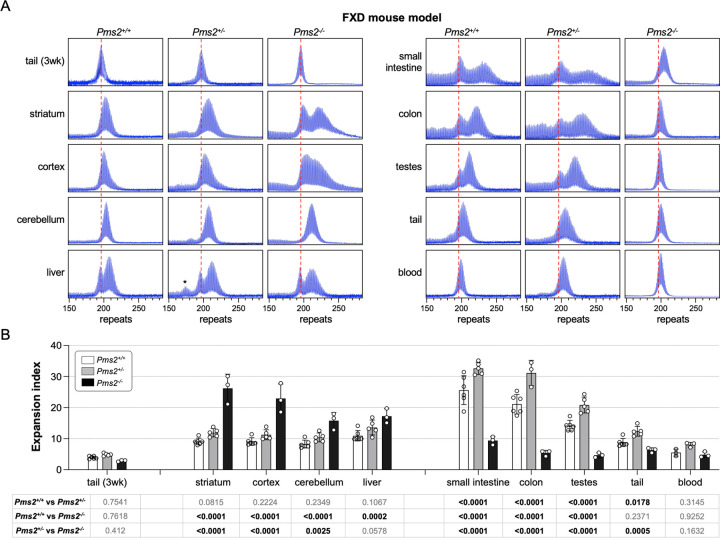
The effect of *Pms2* deficiency on repeat expansion in different tissues of an FXD mouse model. (A) Representative repeat PCR profiles from tail DNA taken at 3 weeks (3 wk) and different organs of 4-month-old *Pms2*^+/+^, *Pms2*^+/−^ and *Pms2*^−/−^ FXD male mice with 196 repeats. The dashed lines represent the sizes of the original inherited alleles as ascertained from the tail DNA taken at 3 weeks. (B) Comparison of the expansion index (EI) in the indicated organs of 4-month-old *Pms2*^+/+^, *Pms2*^+/−^ and *Pms2*^−/−^ FXD mice with an average of 194 repeats in the original allele. The colon data represent the average of 6 *Pms2*^+/+^, 3 *Pms2*^+/−^ and 3 *Pms2*^−/−^ mice with 185–210 repeats. The blood data represent the average of 3 *Pms2*^+/+^, 3 *Pms2*^+/−^ and 3 *Pms2*^−/−^ mice in the same repeat range. The data from other organs represents the average of 6 *Pms2*^+/+^, 5 *Pms2*^+/−^ and 3 *Pms2*^−/−^ mice in the same repeat range. The error bars indicate the standard deviations of the mean. Each dot represents one animal. In each organ, the EIs for different genotypes were compared using a two-way ANOVA with correction for multiple testing as described in the Materials and Methods. The adjusted P-values are listed in the table below. The asterisks in the *Pms2*^+/−^ liver sample indicates a contracted allele that is also present in other organs and not a specific contraction caused by PMS2 deficiency.

To examine the role of PMS2 in repeat expansion in an FXD mouse model, we crossed FXD mice to mice with a null mutation in *Pms2*. We then examined the expansion profiles in animals matched for age and repeat number. As can be seen in Fig. 1, heterozygosity for *Pms2* results in an increase in expansions in most, if not all, expansion-prone tissues including striatum, liver and small intestine. This would be consistent with the interpretation that PMS2 normally protects against expansion. In *Pms2*^−/−^ mice, a further increase in expansions was seen in some tissues, including the striatum, cortex, cerebellum and liver, that would be consistent with this interpretation. However, in other organs including small intestine, colon and blood, fewer expansions were seen. This would suggest that in some circumstances PMS2 plays an important role in promoting expansion as had been observed previously in different model systems^11,20^.

### PMS2 also plays a similar dual role in somatic expansion in an HD mouse model.

**Fig.2.**
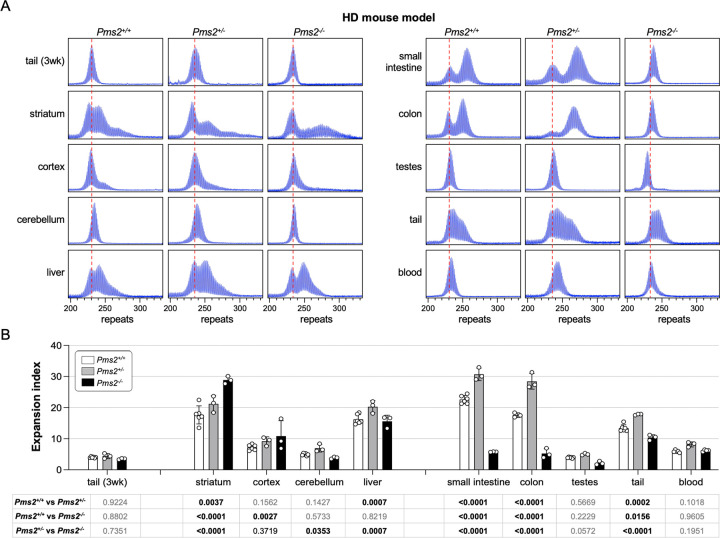
The effect of *Pms2* deficiency on repeat expansion in different tissues from an HD mouse model. (A) Representative repeat PCR profiles from tail DNA taken at 3 weeks (3 wk) and different organs of 4-month-old *Pms2*^+/+^, *Pms2*^+/−^ and *Pms2*^−/−^ HD male mice with ~230 repeats. The dashed lines represent the sizes of the original inherited alleles as ascertained from the tail DNA taken at 3 weeks. (B) Comparison of the expansion index (EI) in the indicated organs of 4-month-old *Pms2*^+/+^, *Pms2*^+/−^ and *Pms2*^−/−^ HD mice with an average of 234 repeats in the original allele. The colon data represent the average of 3 *Pms2*^+/+^, 3 *Pms2*^+/−^ and 3 *Pms2*^−/−^ mice with 226–239 repeats. The data from other organs represents the average of 6 *Pms2*^+/+^, 3 *Pms2*^+/−^ and 3 *Pms2*^−/−^ mice in the same repeat range. The error bars indicate the standard deviations of the mean. Each dot represents one animal. In each organ, the EIs for different genotypes were compared using a two-way ANOVA with correction for multiple testing as described in the Materials and Methods. The adjusted P-values are listed in the table below.

To assess the role of PMS2 in repeat expansion in an HD mouse model, we crossed HD mice to the same *Pms2* null mice and again assessed repeat instability in animals matched for age and repeat number. Consistent with FXD mouse model, in the HD mouse model, heterozygosity for *Pms2* results in an increase in expansions in most expansion-prone tissues (Fig. 2). Furthermore, striatum, which showed more extensive expansions in FXD mice nullizygous for *Pms2*, also showed a similar increase in the extent of expansion in the HD mice. Some organs like small intestine and colon that showed fewer expansions in the FXD mice also showed fewer expansions in the HD mice.

### Exceptions to the rule

While the observations discussed above are consistent with PMS2 playing a similar paradoxical role in affecting the stability of both the FXD and HD repeats in many organs or cell types, some differences are seen in the *Pms2* nullizygous mice. For example, more expansions of the FXD repeat were seen in the cerebellum and liver of *Pms2*^−/−^ mice relative to *Pms2*^+/−^ mice, whereas fewer expansions of the HD repeat were seen in these organs.

**Fig.3.**
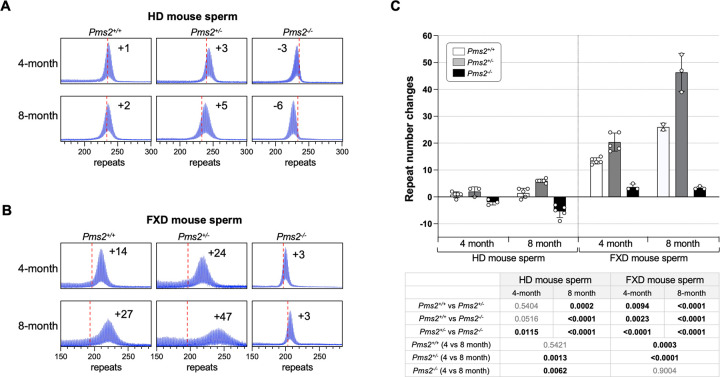
The effect of *Pms2* deficiency on repeat instability in sperm from HD and FXD mouse models. (A) Representative repeat PCR profiles from 4- and 8-month-old *Pms2*^+/+^, *Pms2*^+/−^ and *Pms2*^−/−^ HD male mice with ~230 repeats. The number associated with each profile indicates the change in repeat number relative to the original inherited allele. The dashed lines represent the sizes of the original inherited alleles as ascertained from the tail DNA taken at 3 weeks. (B) Representative repeat PCR profiles from 4- and 8-month-old *Pms2*^+/+^, *Pms2*^+/−^ and *Pms2*^−/−^ FXD male mice with ~197 repeats. The number associated with each profile indicates the change in repeat number relative to the original inherited allele. The dashed lines represent the sizes of the original inherited alleles as ascertained from the tail DNA taken at 3 weeks. (C) Comparison of the repeat number changes in the sperm of 4- and 8-month-old *Pms2*^+/+^, *Pms2*^+/−^ and *Pms2*^−/−^ mice. The 4-month-old HD mice data represents the average of 5 *Pms2*^+/+^, 3 *Pms2*^+/−^ and 3 *Pms2*^−/−^ mice with 229–239 repeats (average of 234 repeats) in the original allele. The 8-month-old HD mice data represents the average of 5 *Pms2*^+/+^, 6 *Pms2*^+/−^ and 5 *Pms2*^−/−^ mice with 219–235 repeats (average of 225 repeats) in the original allele. The 4-month-old FXD mice data represents the average of 5 *Pms2*^+/+^, 5 *Pms2*^+/−^ and 3 *Pms2*^−/−^ mice with 185–210 repeats (average of 193 repeats) in the original allele. The 8-month-old FXD mice data represents the average of 2 *Pms2*^+/+^, 3 *Pms2*^+/−^ and 3 *Pms2*^−/−^ mice with 197–224 repeats (average of 209 repeats) in the original allele. The error bars indicate the standard deviations of the mean. Each dot represents one animal. In each mouse model, the repeat number changes with different genotype and age were compared using a two-way ANOVA with correction for multiple testing as described in the Materials and Methods. The adjusted P-values are listed in the table below.

Testes was another organ in which differences in the response to the loss of PMS2 were seen (Fig. 2). Expansions in the FXD testes of *Pms2*^+/+^ mice were extensive, while they were relatively modest in the testes of *Pms2*^+/+^ HD mice. This is consistent with previous work showing that FXD repeat expansions in mouse testes occurs primarily in the spermatogonial stem cell (SSC) pool^25^, whereas HD expansions in both mice and humans occur later in spermatogenesis^26–28^. As a result, the repeat number in sperm increases significantly over time in FXD mice but not in HD mice, likely due to accumulation of FXD repeat expansions in SSCs. Despite this difference, as in other expansion-prone tissues, FXD and HD repeats in testes expanded faster in *Pms2*^+/−^ mice than in *Pms2*^+/+^ mice. What was surprising, however, was the size of the HD repeats in the testes of *Pms2*^−/−^ mice. Unlike the FXD repeats in *Pms2*^−/−^ testes, where the modal allele was slightly larger than the modal allele in tail taken at weaning, the HD repeat number in *Pms2*^−/−^ testes was actually smaller than it was in the initial inherited allele, consistent with contractions. To study this phenomenon in more detail, we compared the change in the modal repeat length in the sperm of *Pms2*^+/+^, *Pms2*^+/−^ and *Pms2*^−/−^ mice at 4 months and 8 months of age. As can be seen in Fig. 3, the repeat number in the sperm of HD mice lacking PMS2 actually decreased with age, while the repeat number remained stable in the FXD mice.

## Discussion

We show here that mice heterozygous for *Pms2* all show more expansion of the FXD and HD repeats in expansion-prone tissue than *Pms2*^+/+^ mice (Fig. 1 and Fig. 2). This would be consistent with PMS2 playing a role in preventing expansion of both repeats. However, while in nullizygous animals, some organs showed a further increase in expansions of both repeats consistent with a protective role for PMS2, other organs showed a significant decrease relative to heterozygous and WT animals demonstrating that in some organs or cell types, PMS2 can be pro-mutagenic and involved in the generation of expansions. With some exceptions, both repeats respond similarly to the absence of PMS2 in expansion-prone organs. Thus, the different effects of PMS2 reported in different model systems of different REDs likely do not reflect fundamentally different mechanisms of instability in different diseases, but rather the fact that PMS2 has paradoxical effects on repeat expansion in general.

**Fig.4.**
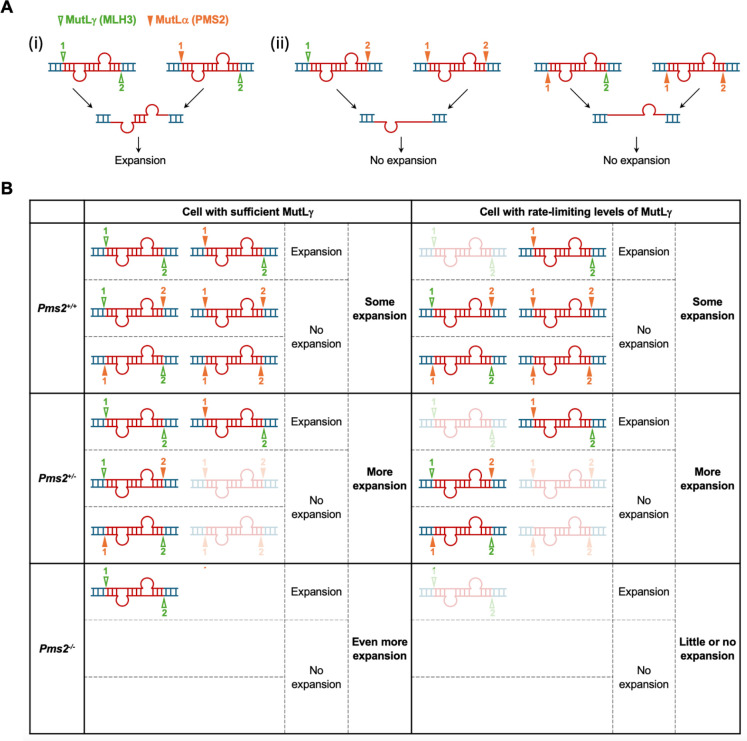
Model for the differential effects of a PMS2 deficiency on the probability of repeat expansion. A double loop-out structure can form in the region of repeats (show in red) when DNA is transiently unpaired. Depending on the relative abundance of different MMR proteins and their relative binding affinities, each loop-out is bound by either MutLγ or MutLα, and then either the same strand or the opposite strand of the loop-out will be cleaved by the MutL complexes. MutLγ always cuts the opposite strand of the loop-out it binds, whereas MutLα will cut the same strand if there is a pre-existing nick. Without a pre-existing nick, MutLα has an equal probability of cutting either strand. (A) Model for the generation of expansion intermediates by differential MutL cleavage. There are six ways to cut the double loop-out, depending on which MutL complex cuts first. The triangles represent cut sites with numbers on the triangles indicating the order of cleavage. Three different intermediates will be generated after cleavage. (i) In the case of intermediates generated by cleavage of different strands, gap-filling the two looped-out regions will result in expansion. (ii) When both cuts occur on the same strand, excision or strand-displacement results in the removal of one loop-out. After gap-filling by Polδ the original allele will be restored (no change). (B) The expansion probabilities in cells with different relative levels of MLH3 and PMS2. In cells with sufficient MLH3, PMS2 deficiency will reduce the possibility of cleavages that required PMS2. Thus, the likelihood of MLH3 making both cleavages will increase, resulting in an increase in expansion. More loop-outs will be cleaved by MLH3 in the absence of PMS2, leading to a higher level of expansion. In cells insufficient in MLH3, PMS2 is required to make the first cut in the presence of MLH3 to generate expansion. However, PMS2 deficiency will reduce the possibility of PMS2 making two cleavages, thus increasing the chance of MLH3 making the second cleavage, which results in increased expansion. Insufficient MLH3 to make two cleavages in the absence of PMS2 will result in little or no expansion. The fainter (or absent) graphic reflects its reduced probability.

PMS2 and MLH1 form the heterodimer MutLα, the major complex involved in lesion processing in mammalian MMR. Previous work in different model systems has demonstrated that MutLγ, a heterodimer of MLH1 and MLH3, is required for expansions^11,29–31^. MLH3 has been shown *in vitro* to cleave the DNA strand opposite any loop-out to which it binds^32^. In contrast, on nicked substrates *in vitro*, PMS2 cleaves the nicked strand, while in the absence of a nick, it has an equal probability of cutting either strand^33–35^. The simplest interpretation of our data would be consistent with these same cleavage preferences *in vivo*. If the expansion substrate were to have two equal sized loop-outs, perhaps resulting from strand misalignment during transcription, cleavage by MutLγ would always result in cuts on opposite strands. These could be processed by exonucleases to generate a pair of offset gaps located opposite each loop-out. Subsequent gap-filling would result in the addition of repeats corresponding to the size of a single loop-out. On the other hand, cleavage by MutLα would have different effects depending on whether MutLγ was also available and which MutL complex cut first. Thus, when MutLγ was available, the loop-outs would sometimes be processed to generate an intermediate with offset gaps on both strands that would result in expansions as illustrated in Fig. 4A–(i). However, at some frequency in the presence of MutLγ, or whenever MutLγ was unavailable, two cuts on the same strand of the loop-out substrate would be generated as illustrated in Fig. 4A–(ii). After exonuclease processing an intermediate with a gap on one strand would result, and gap-filling of this intermediate could restore the original allele. Thus, the extent of expansion in a particular cell would depend on the relative levels of the two MutL complexes as illustrated in Fig. 4B. Thus this model may provide the first *in vivo* support for the MutLα and MutLγ cleavage preferences determined *in vitro*^32–35^.

While in many tissues the FXD and the HD repeats respond to *Pms2* gene dosage in similar ways, there are some differences. For example, while heterozygosity for *Pms2* results in an increase in expansions of both the FXD and the HD repeat in testes, nullizygosity results in a decrease in FXD repeat expansions relative to heterozygous and WT animals, while the HD repeat instead contracts. Since the repeat size of the FXD repeat seen in sperm from 4- and 8-month-old animals remains stable (Fig. 3), this decrease is not progressive. This differs from organs like the small intestine where expansions in the nullizygous animals are reduced but continue to accumulate with age (Fig. S1). We speculate that in *Pms2*^−/−^ animals a small amount of expansion of the FXD repeat occurs prenatally in a precursor cell population where PMS2 is protective, with the failure to expand postnatally in the testes reflecting a requirement for PMS2 for expansion in the SSC pool. In contrast, the HD contractions seen in the *Pms2*^−/−^ sperm may reflect classical microsatellite instability related to an MMR deficiency as we saw previously in FXD mESCs and GLSD iPSCs^11,20^. Differences are also seen in the cerebellum and liver of *Pms2* null mice where there are more expansions relative to *Pms2*^+/−^ mice for the FXD repeat, but fewer expansions for the HD repeat. Whether this reflects small differences in the relative levels of expansion substrate remains to be seen.

Nonetheless, our data support the idea that the differences in the effects observed for PMS2 in different model systems do not reflect fundamental differences in the expansion mechanism in these models or in the REDs themselves. This has implications for our understanding of the mechanisms controlling repeat instability in this group of disorders. It also increases the confidence that a successful approach for reducing somatic expansions in one of these diseases will be useful to the other diseases in this group.

## Materials and Methods

### Reagents and services

Reagents were from Sigma-Aldrich (St Louis, MO, USA) unless otherwise stated. Primers were from Life Technologies (Grand Island, NY, USA). Capillary electrophoresis of fluorescently labeled PCR products was carried out by the Roy J Carver Biotechnology Center, University of Illinois (Urbana, IL, USA) and Psomagen (Rockville, MD).

### Mouse generation, breeding, and maintenance

Embryos of *Pms2* mutant mice^36^ were obtained from The Jackson Laboratory (Bar Harbor, ME; JAX stock #010945) and recovered by NIDDK Laboratory Animal Sciences section (LASS) using standard procedures. The FXD mice^37^ and HD mice^38^ have been previously described. *Pms2* mutant mice were crossed to FXD and HD mice to generate animals that were heterozygous for *Pms2*. These mice were then crossed again with FXD or HD mice to generate mice homozygous for the *Pms2* mutation. All mice were on a C57BL/6J background. Mice were maintained in a manner consistent with the Guide for the Care and Use of Laboratory Animals (NIH publications no. 85–23, revised 1996) and in accordance with the guidelines of the NIDDK Animal Care and Use Committee, who approved this research (ASP-K021-LMCB-21).

### DNA isolation

DNA for genotyping was extracted from mouse tails collected at 3-weeks-old, or weaning, using KAPA Mouse Genotyping Kit (KAPA Biosystems, Wilmington, MA). DNA was isolated from a variety of tissues that were collected from 4- and 8-month-old male mice using a Maxwell^®^ 16 Mouse Tail DNA Purification kit (Promega, Madison, WI) according to the manufacturer’s instructions. A 5 cm section of the jejunum was collected as the small intestine sample and a 5 cm distal colon sample was collected upstream of the anus as previously described^39^. Sperm collection and DNA preparation were as previously described^40^.

### Genotyping and analysis of repeat number

Genotyping of *Pms2* was carried out using the KAPA mouse genotyping kit (KAPA Biosystems) according to manufacturer’s instructions with primers JAX-9366 (5’-TTCGGTGACAGATTTGTAAATG-3’) and JAX-9367 (5’-TCACCATAAAAATAGTTTCCCG-3’) used to detect the WT *Pms2* allele and JAX-9366 and JAX-9368 (5’-TTTACGGAGCCCTGGC-3’) to detect the mutant *Pms2* allele. The PCR mix for the *Pms2* allele contained 2 uL template DNA, 1X KAPA2G Fast HotStart Genotyping Mix (KAPA Biosystems, Wilmington, MA, USA), and 0.5 μM each of the primers. The *Pms2* allele PCR conditions were 95°C for 3 min; 35 cycles of 95°C for 15 s, 60°C for 15 s and 72°C for 15 s; followed by 72°C for 3 min. Genotyping and repeat size analysis of the *Fmr1* and *Htt* alleles was performed using a fluorescent PCR assay with fluorescein amidite (FAM)-labeled primer pairs. The primers FAM-labeled FraxM4 (FAM-5’-CTTGAGGCCCAGCCGCCGTCGGCC-3’) and FraxM5 (5’-CGGGGGGCGTGCGGTAACGGCCCAA-3’) were used for the *Fmr1* allele^37^. The PCR mix for *Fmr1* allele contained 3 uL (150 ng) template DNA, 1X KAPA2G Fast HotStart Genotyping Mix (KAPA Biosystems, Wilmington, MA, USA), 2.4 M betaine, 2% DMSO, 0.5 μM each of the primers and additional of 125 μM each of dCTP and dGTP. The PCR cycling parameters for the *Fmr1* allele were 95°C for 10 min; 35 cycles of 95°C for 30 s, 65°C for 30 s and 72°C for 90 s; followed by 72°C for 10 min. The primers FAM-labeled HU3 (FAM-5’-GGCGGCTGAGGAAGCTGAGGA-3’) and Htt-EX1-F1 (5’-GCAACCCTGGAAAAGCTGATGAAGGC-3’) were used for the *Htt* allele. The PCR mix for the *Htt* allele contained 2 μL (100 ng) DNA template, 1x KAPA2G Fast HotStart Genotyping Mix (KAPA Biosystems, Wilmington, MA, USA), 1.2 M betaine, 1% DMSO, and 0.5 μM each of the primers. The *Htt* allele was amplified by touchdown PCR using the following parameters: 95°C for 10 min; 10 cycles of 95°C for 30 s, 72°C with −1°C/cycle for 30 s and 72°C for 90 s; 28 cycles of 95°C for 30 s, 63°C for 30 s and 72°C for 90 s; followed by 72°C for 10 min. The *Fmr1* and *Htt* PCR products were resolved by capillary electrophoresis on an ABI Genetic Analyzer and the resultant fsa files were displayed using a previously described custom R script^41^ that is available upon request.

The original inherited allele repeat size was indicated by the tail sample that was taken at 3-weeks or weaning of the mice. The expansion index (EI) was calculated in the same way as the somatic instability index^42^, but only peaks larger than the original inherited allele were considered, with a cutoff of 10% relative peak height threshold. The repeat number changes were determined by subtracting the number of repeats in the modal allele from the number of repeats in the original inherited allele.

### Statistical analyses

Statistical analyses were performed using GraphPad Prism 10.2. For comparisons of EI or repeat number changes in samples with different genotypes or ages, statistical significance was assessed using the two-way ANOVA with Tukey’s multiple comparisons correction.

## Supplementary Material

Supplement 1

## Data Availability

All data generated or analyzed during this study are included in this published article and its Supplementary Information files.
