# PMS2 has both pro-mutagenic and anti-mutagenic effects on repeat instability in the Repeat Expansion Diseases

**DOI:** 10.1101/2024.08.13.607839

**Published:** 2025-04-02

**Authors:** Diego Antonio Jimenez, Carson J. Miller, Alexandra Walker, Kusala Anupindi, Karen Usdin, Xiaonan Zhao

**Affiliations:** 1 Section on Gene Structure and Disease, Laboratory of Cell and Molecular Biology, National Institute of Diabetes and Digestive and Kidney Diseases, National Institutes of Health, Bethesda, MD 20892

**Keywords:** microsatellite instability, MutLα, MutLγ, Huntington’s disease, fragile X-related disorders

## Abstract

Expansion of a disease-specific tandem repeat is responsible for >45 Repeat Expansion Diseases (REDs). The mismatch repair protein PMS2 is a modifier of somatic expansion and disease severity in Huntington’s disease (HD), a RED resulting from a CAG-repeat expansion. However, PMS2 shows different effects in different RED models, protecting against expansion in some and promoting it in others. To better understand this difference, we carried out a systematic study of the loss of PMS2 in mouse models of HD and the fragile X-related disorders (FXDs), a group of REDs resulting from a CGG-repeat expansion. In both models, loss of one *Pms2* allele resulted in more expansions, while loss of both alleles resulted in more expansion in some organs but less in others. Thus, rather than reflecting different expansion mechanisms in different diseases, the previously reported differences in different model systems likely reflects the ability of PMS2 to promote expansion in some cellular contexts and to protect against it in others. In mouse embryonic stem cells containing both sets of repeats where PMS2 was expressed under the control of a doxycycline (DOX)-inducible promoter, low DOX concentrations produced a dose-dependent increase in expansions of both repeats, an effect that was dependent on the PMS2 nuclease domain, while higher DOX levels resulted in a decrease in expansions. Our findings have implications both for the mechanism of expansion and for therapeutic approaches to treat these diseases by reducing somatic expansion.

## Significance statement

Expansion of a disease-specific microsatellite is responsible for >45 Repeat Expansion Diseases (REDs). The mismatch repair factor PMS2 has been identified as a genetic modifier of somatic expansion and disease severity in Huntington’s disease (HD). However, loss of PMS2 has different effects in different REDs models. Using mouse and cell models of the fragile X-related disorders and HD, we show that PMS2 has both pro-mutagenic and anti-mutagenic effects in both models depending on the cell type and that an intact nuclease domain is required for its ability to promote expansion. These findings support the idea that the REDs all share a common expansion mechanism and suggests a model for repeat expansion consistent with a dual role for PMS2 in this process.

## Introduction

Repeat expansion, the increase in the number of repeats in a short tandem repeat (STR) in a disease-specific gene, is the cause of the Repeat Expansion Diseases (REDs), a group of >45 life-limiting neurological or neurodevelopmental disorders (1) with a collective allele frequency of 1 in 283 individuals (2). Converging evidence from studies of genetic modifiers in patients with different REDs implicates components of the Mismatch Repair (MMR) pathway as modifiers of both repeat expansion and disease severity (3–7). This has raised the possibility that targeting some of these factors may be useful therapeutically (8), an appealing idea since these diseases currently have no effective treatment or cure. Furthermore, if indeed these diseases share a common mechanism, a single treatment may be useful for multiple diseases in this group. Some of the same factors identified as modifiers of somatic expansion in human studies have been implicated in expansion in different cell and mouse models of these disorders. For example, MLH3, the binding partner of MLH1 in the MutLγ complex involved in lesion processing in MMR, is required for expansion in multiple disease models (9–16). Taken together these findings suggest that different REDs may expand using the same or very similar mechanisms and that these cell and mouse models are suitable for understanding the expansion process in humans.

However, discordant effects have been seen for some of the MMR factors in different diseases and disease models, casting doubt on the idea that all REDs share a common mechanism or, at least, suggesting that there are important differences between diseases for some important genetic modifiers. For example, PMS2, the MLH1 binding partner in MutLα, another lesion processing complex in MMR, promotes repeat expansion in some models but protects against it in others. Genome wide association studies (GWAS) have shown that PMS2 is a modifier of age at onset and risk of somatic expansion in Huntington’s disease (HD) (17, 18), a CAG-repeat expansion disorder (19). Somatic expansion in HD has been linked to modifier haplotypes that are associated with both earlier and later onset (6, 18). PMS2 has also been shown to protect against expansion in the brain (20, 21) and liver (20) of HD mouse models as well as in the brain of a mouse model of Friedreich’s ataxia (FRDA) (22), a GAA-repeat expansion disorder (23). However, PMS2 was shown to be required for expansion in mouse embryonic stem cells (mESCs) from a mouse model of the Fragile X-related disorders (FXDs) (24), disorders caused by CGG-repeat expansion in the Fragile X Messenger Ribonucleoprotein 1 (*FMR1*) gene (25). This requirement for PMS2 was also observed in induced pluripotent stem cells (iPSCs) derived from a patient with Glutaminase Deficiency (GLSD) (12), a CAG-repeat expansion disorder (26). Furthermore, in a mouse model of Myotonic Dystrophy Type 1 (DM1), a CTG-repeat expansion disorder (27), loss of PMS2 results in the loss of ~50% of expansions in many organs (28).

To understand these differences, we carried out a systematic comparison of the effect of the loss of PMS2 in different organs of two different REDs mouse models: a mouse model of FXDs and a mouse model of HD. Using age-matched animals with similar repeat numbers for each model, we found that in both models PMS2 had contrasting effects: promoting expansion in some cell types and suppressing it in others. We also showed that expansion rates for both repeats peak and then decline with increasing PMS2 dosage in a mouse embryonic stem cell (mESC) model, with PMS2’s ability to promote expansion depending on its nuclease domain. Our findings provide insight into the nature of the expansion substrate, the expansion mechanism itself, and provide insights into strategies to target components of the MMR pathway to reduce somatic expansion.

## Results

### PMS2 plays a dual role in somatic expansion in an FXD mouse model.

**Fig. 1.**
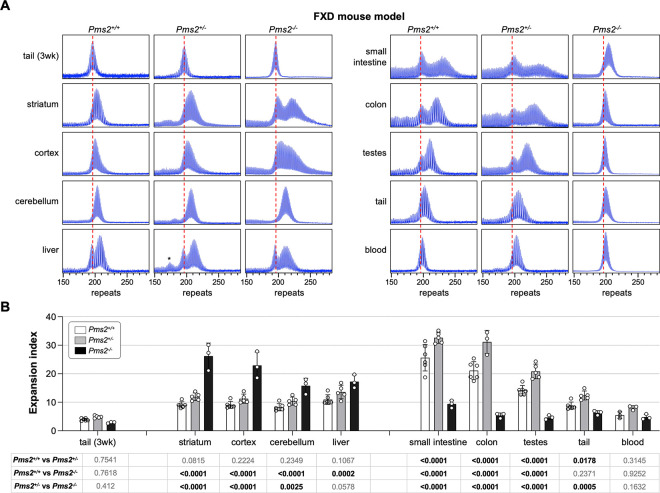
The effect of *Pms2* deficiency on repeat expansion in different tissues of an FXD mouse model. (A) Representative repeat PCR profiles from tail DNA taken at 3 weeks (3 wk) and different organs of 4-month-old *Pms2*^+/+^, *Pms2*^+/−^ and *Pms2*^−/−^ FXD male mice with 196 repeats. The dashed lines represent the sizes of the original inherited alleles as ascertained from the tail DNA taken at 3 weeks. (B) Comparison of the expansion index (EI) in the indicated organs of 4-month-old *Pms2*^+/+^, *Pms2*^+/−^ and *Pms2*^−/−^ FXD mice with an average of 194 repeats in the original allele. The colon data represent the average of 6 *Pms2*^+/+^, 3 *Pms2*^+/−^ and 3 *Pms2*^−/−^ mice with 185–210 repeats. The blood data represent the average of 3 *Pms2*^+/+^, 3 *Pms2*^+/−^ and 3 *Pms2*^−/−^ mice in the same repeat range. The data from other organs represents the average of 6 *Pms2*^+/+^, 5 *Pms2*^+/−^ and 3 *Pms2*^−/−^ mice in the same repeat range. The error bars indicate the standard deviations of the mean. Each dot represents one animal. In each organ, the EIs for different genotypes were compared using a two-way ANOVA with correction for multiple testing as described in the Materials and Methods. The adjusted P-values are listed in the table below. The asterisks in the *Pms2*^+/−^ liver sample indicates a contracted allele that is also present in other organs and not a specific contraction caused by PMS2 deficiency.

To examine the role of PMS2 in repeat expansion in an FXD mouse model, we crossed FXD mice to mice with a null mutation in *Pms2*. We then examined the expansion profiles in animals matched for age and repeat number. As can be seen in Fig. 1, heterozygosity for *Pms2* results in an increase in expansion in most, if not all, expansion-prone tissues including striatum, liver and small intestine. This would be consistent with the interpretation that PMS2 normally protects against expansion. In *Pms2*^−/−^ mice, a further increase in expansion was seen in some tissues, including the striatum, cortex, cerebellum, and liver, that would also be consistent with this interpretation. However, in other organs, including small intestine, colon and blood, fewer expansions were seen in *Pms2* null mice than were seen in heterozygous mice. Thus, in the very same animals, PMS2 can both promote expansion and prevent it depending on cell type.

### PMS2 plays a similar dual role in somatic expansion in an HD mouse model.

**Fig. 2.**
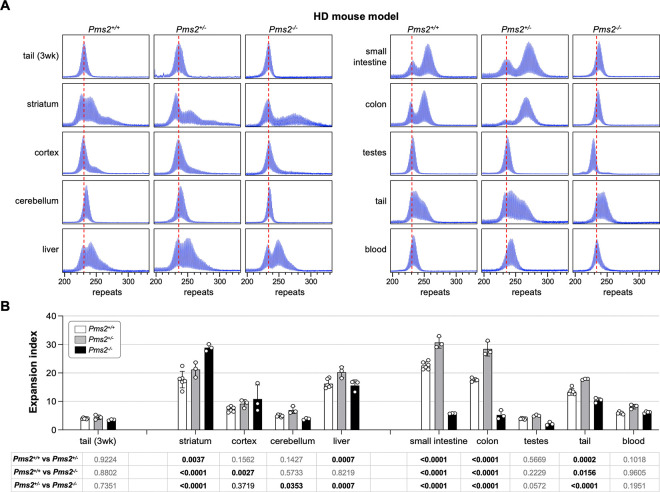
The effect of *Pms2* deficiency on repeat expansion in different tissues from an HD mouse model. (A) Representative repeat PCR profiles from tail DNA taken at 3 weeks (3 wk) and different organs of 4-month-old *Pms2*^+/+^, *Pms2*^+/−^ and *Pms2*^−/−^ HD male mice with ~230 repeats. The dashed lines represent the sizes of the original inherited alleles as ascertained from the tail DNA taken at 3 weeks. (B) Comparison of the expansion index (EI) in the indicated organs of 4-month-old *Pms2*^+/+^, *Pms2*^+/−^ and *Pms2*^−/−^ HD mice with an average of 234 repeats in the original allele. The colon data represent the average of 3 *Pms2*^+/+^, 3 *Pms2*^+/−^ and 3 *Pms2*^−/−^ mice with 226–239 repeats. The data from other organs represents the average of 6 *Pms2*^+/+^, 3 *Pms2*^+/−^ and 3 *Pms2*^−/−^ mice in the same repeat range. The error bars indicate the standard deviations of the mean. Each dot represents one animal. In each organ, the EIs for different genotypes were compared using a two-way ANOVA with correction for multiple testing as described in the Materials and Methods. The adjusted P-values are listed in the table below.

To assess the role of PMS2 in repeat expansion in an HD mouse model, we crossed HD mice to the same *Pms2* null mice and again assessed repeat instability in animals matched for age and repeat number. Consistent with what was seen in the FXD mouse model, in the HD mouse model, heterozygosity for *Pms2* results in an increase in expansions in most expansion-prone tissues (Fig. 2). Furthermore, striatum, which showed more extensive expansions in FXD mice nullizygous for *Pms2*, also showed a similar increase in the extent of expansion in the HD mice. Interestingly, in animals WT for *Pms2*, a population of cells in the striatum had alleles that were smaller than the allele seen in the tail DNA taken at weaning. The fraction of contracted cells decreased with decreasing *Pms2* gene dosage suggesting that the contractions are PMS2-dependent.

However, as was seen in the FXD mice, in other organs loss of all PMS2 resulted in a decrease rather than in increase in expansions. In contrast to the FXD mouse, this was also the case in liver and cerebellum. Furthermore, the increase of expansion in the striatum and cortex of the *Pms2* null HD mice were smaller than in the *Pms2* null FXD mice, with the difference between heterozygous and nullizygous mice not reaching statistical significance for the cortex. Another difference between the mouse models was seen in the testes. While expansions in the FXD testes of *Pms2*^+/+^ mice were extensive, they were relatively modest in the testes of *Pms2*^+/+^ HD mice. This is consistent with previous work showing that FXD repeat expansions in mouse testes occurs primarily in the spermatogonial cells (SPGs) (29), whereas HD expansions in both mice and humans occur in the spermatozoa (30–32). Since the spermatogonia are the reservoir of replicating cells from which mature gametes are derived, while spermatozoa are a short-lived cell type, successive expansions can accumulate over time in SPGs but not in spermatozoa. This may explain why the repeat number seen in mature sperm increases significantly over time in FXD mice but not in HD mice. Despite this difference, as in other expansion-prone tissues, FXD and HD repeats in testes expanded faster in *Pms2*^+/−^ mice than in *Pms2*^+/+^ mice. However, unlike the FXD repeats in *Pms2*^−/−^ testes where the modal allele was slightly larger than the modal allele in tail taken at weaning, the HD repeat number in *Pms2*^−/−^ testes was smaller than it was in the initial inherited allele, consistent with contractions.

**Fig. 3.**
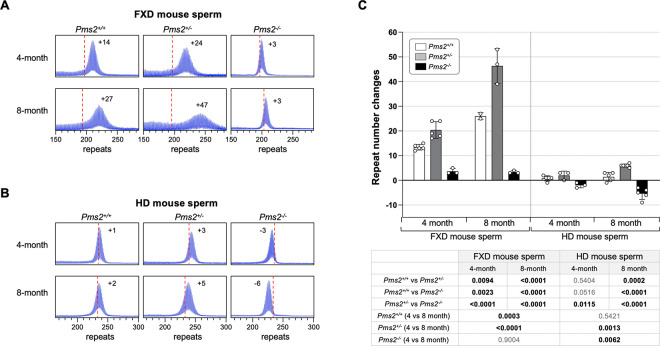
The effect of *Pms2* deficiency on repeat instability in sperm from HD and FXD mouse models. (A) Representative repeat PCR profiles from 4- and 8-month-old *Pms2*^+/+^, *Pms2*^+/−^ and *Pms2*^−/−^ HD male mice with ~230 repeats. The number associated with each profile indicates the change in repeat number relative to the original inherited allele. The dashed lines represent the sizes of the original inherited alleles as ascertained from the tail DNA taken at 3 weeks. (B) Representative repeat PCR profiles from 4- and 8-month-old *Pms2*^+/+^, *Pms2*^+/−^ and *Pms2*^−/−^ FXD male mice with ~197 repeats. The number associated with each profile indicates the change in repeat number relative to the original inherited allele. The dashed lines represent the sizes of the original inherited alleles as ascertained from the tail DNA taken at 3 weeks. (C) Comparison of the repeat number changes in the sperm of 4- and 8-month-old *Pms2*^+/+^, *Pms2*^+/−^ and *Pms2*^−/−^ mice. The 4-month-old HD mice data represents the average of 5 *Pms2*^+/+^, 3 *Pms2*^+/−^ and 3 *Pms2*^−/−^ mice with 229–239 repeats (average of 234 repeats) in the original allele. The 8-month-old HD mice data represents the average of 5 *Pms2*^+/+^, 6 *Pms2*^+/−^ and 5 *Pms2*^−/−^ mice with 219–235 repeats (average of 225 repeats) in the original allele. The 4-month-old FXD mice data represents the average of 5 *Pms2*^+/+^, 5 *Pms2*^+/−^ and 3 *Pms2*^−/−^ mice with 185–210 repeats (average of 193 repeats) in the original allele. The 8-month-old FXD mice data represents the average of 2 *Pms2*^+/+^, 3 *Pms2*^+/−^ and 3 *Pms2*^−/−^ mice with 197–224 repeats (average of 209 repeats) in the original allele. The error bars indicate the standard deviations of the mean. Each dot represents one animal. In each mouse model, the repeat number changes with different genotype and age were compared using a two-way ANOVA with correction for multiple testing as described in the Materials and Methods. The adjusted P-values are listed in the table below.

To study this phenomenon in more detail, we compared the change in the modal repeat length in the sperm of *Pms2*^+/+^, *Pms2*^+/−^ and *Pms2*^−/−^ mice at 4 months and 8 months of age. As can be seen in Fig. 3, while the repeat number remained stable in the sperm of FXD mice lacking PMS2, it decreased with age in the sperm of HD mice. Thus, contractions occurring in the HD repeats in the testes are different from the contractions observed for the HD repeats in the striatum in that they are not PMS2-dependent. Rather they resemble the typical microsatellite instability associated with an MMR deficiency as we previously observed in stem cell models of different REDs (7, 24).

### PMS2 is required for expansion of both FXD and HD repeats in mESCs.

**Fig. 4.**
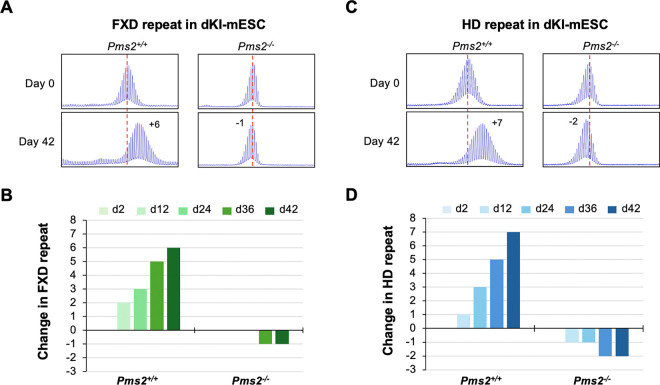
Effect of *Pms2* deficiency on expansion of FXD and HD repeats in a double knock-in mESC model. (A, C) Repeat PCR profiles of FXD (A) and HD (C) repeats in *Pms2*^+/+^ and *Pms2*^−/−^ dKI-mESCs carrying both FXD and HD repeats. The numbers in the day 42 profiles indicate the change in repeat number. The red dotted line indicates the starting allele. (B, D) Changes in FXD (B) and HD (D) repeats number at the indicated days (d) in culture.

To study both repeats in the same system we generated double knock-in (dKI) mESCs carrying ~180 FXD repeats and ~215 HD repeats. These lines showed similar rates of expansion as single knock-in cell lines (Fig. S1). We then used CRISPR-Cas9 to knockout *Pms2* in a dKI-mESC line. No expansion of either repeat was seen in these lines (Fig. 4). Thus, PMS2 is required for expansions of both repeats in this model system. This is consistent with our previous demonstration for the FXD repeat (24) as well as the CAG repeat responsible for GLSD (7). As we had previously observed, complete loss of PMS2 resulted in small decreases in repeat number for both repeats.

**Fig. 5.**
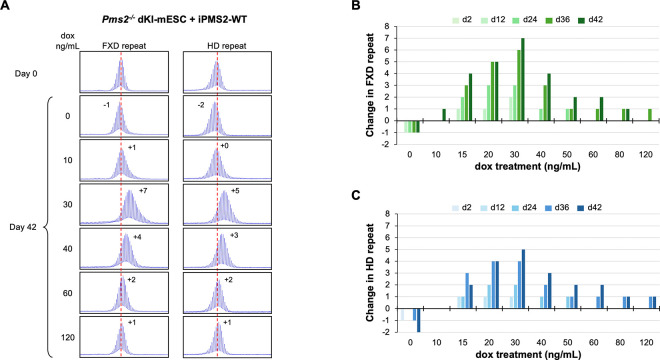
Effect of doxycycline-induced WT PMS2 expression on expansion of FXD and HD repeats in a double knock-in mESC model. (A) Repeat PCR profiles of FXD (left) and HD (right) repeats in *Pms2*^−/−^ dKI-mESCs expressing DOX-induced WT PMS2 (iPMS2-WT) at different concentrations of DOX after 42 days in culture. The number associated with each profile indicates the change in repeat number. The red dotted line indicates the starting allele. DOX concentrations producing similar levels of both DOX-induced WT and D696N versions of the DOX-induced PMS2 protein were used. (B, C) Changes in FXD (B) and HD (C) repeat number over time in culture in *Pms2*^−/−^ dKI-mESCs expressing DOX-induced WT PMS2 at different concentrations of DOX. Days in culture (d) indicated above graphs.

**Fig. 6.**
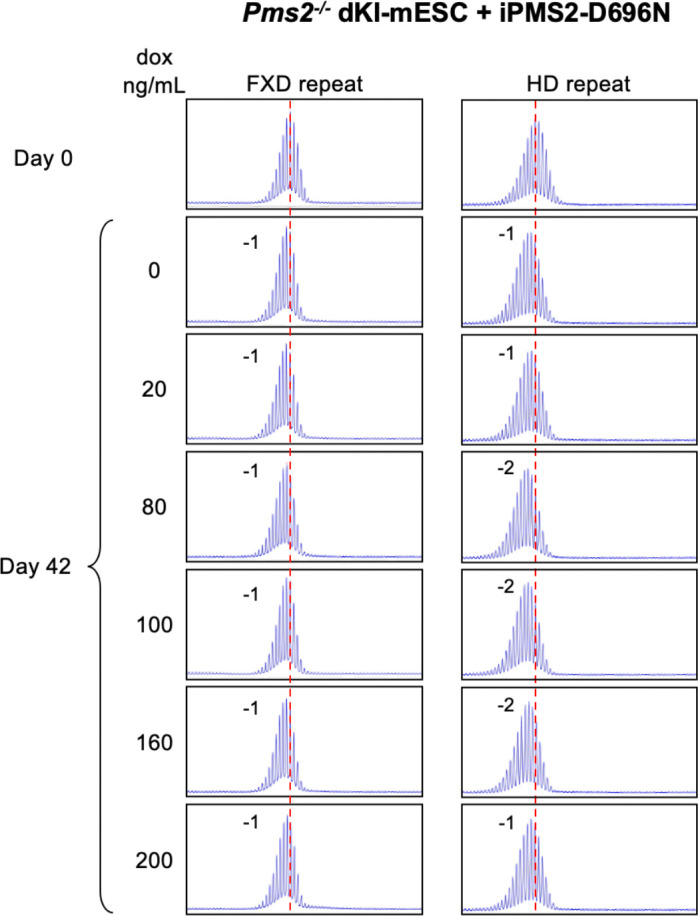
Effect of doxycycline-induced PMS2-D6G6N expression on expansion of FXD and HD repeats in a double knock-in mESC. (A) Repeat PCR profiles of FXD (left) and HD (right) repeats in *Pms2*^−/−^ dKI-mESCs expressing DOX-induced PMS2 D696N (iPMS2-D696N) at different concentrations of DOX after 42 days in culture. The number associated with each profile indicates the change in repeat number. The red dotted line indicates the starting allele. DOX concentrations producing similar levels of both DOX-induced WT and D696N versions of the DOX-induced PMS2 protein were used.

To test the effect of PMS2 dosage on expansion, we integrated a doxycycline (DOX)-inducible WT PMS2-expressing construct into a *Pms2*^−/−^ dKI-mESC line and monitored the stability of both the FXD and the HD repeats over time in different concentrations of DOX. Without DOX treatment, small contractions like those seen in *Pms2*^−/−^ lines were also seen (Fig. 5). As the concentration of DOX was increased, so a progressive increase in repeat expansions was seen for both repeats reaching a maximum at 30 ng/mL. The extent of expansions at this DOX concentration was similar to the extent of expansions seen in *Pms2*^+/+^ cells (Fig. 4). Higher concentrations of DOX resulted in a progressive decrease in expansions of both repeats. Thus, both too little PMS2 and too much can result in decreased expansion. However, in contrast to the contractions seen when PMS2 is lost, in the case of overexpression of PMS2 some expansions were still seen after extended growth at the highest DOX concentrations with no evidence of contractions. We repeated this experiment with a construct expressing similar levels of PMS2 containing a D696N mutation in the nuclease domain (Fig. S2). As can be seen in Fig. 6, only small contractions were seen for both repeats at all DOX concentrations tested. Thus, an intact nuclease domain is required for PMS2’s effect on expansion.

## Discussion

We show here that mice heterozygous for *Pms2* show more expansion of the FXD and HD repeats in expansion-prone tissue than *Pms2*^+/+^ mice (Fig. 1 and Fig. 2). This is consistent with PMS2 playing a role in preventing expansion of both the FXD and the HD repeats. However, in nullizygous animals, while some organs showed a further increase in expansions of both repeats consistent with a protective role for PMS2, other organs showed a significant decrease relative to heterozygous and WT animals. Thus, in some organs or cell types, PMS2 promotes expansions *i.e.*, it can act pro-mutagenically. This idea is substantiated by our demonstration in mESCs that loss of PMS2 eliminates expansions of both the FXD and the HD repeats (Fig. 4). Further, by experimentally increasing levels of PMS2 in a *Pms2*^−/−^ cell line, expansion rates rise and then fall, peaking at expansion rates similar to those seen in WT cells for both repeats (Fig. 5). Therefore, the different effects of PMS2 reported in different model systems of different REDs likely do not reflect fundamentally different mechanisms of instability in these diseases, but rather the fact that PMS2 has different effects on repeat expansion in different cell types.

**Fig. 7.**
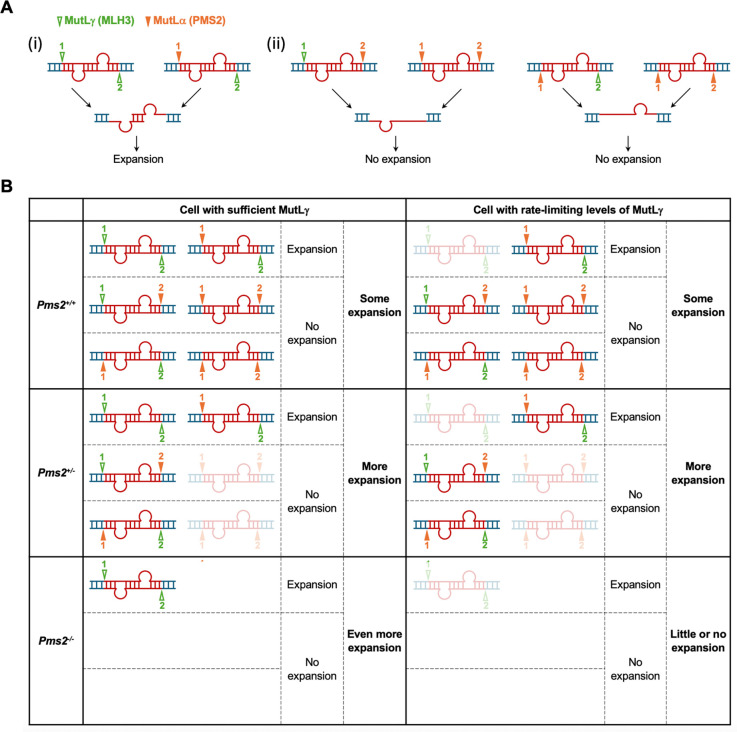
Model for the differential effects of a PMS2 deficiency on the probability of repeat expansion. A double loop-out structure can form in the region of repeats (show in red) when DNA is transiently unpaired. Depending on the relative abundance of different MMR proteins and their relative binding affinities, each loop-out is bound by either MutLγ or MutLα, and then either the same strand or the opposite strand of the loop-out will be cleaved by the MutL complexes. MutLγ always cuts the opposite strand of the loop-out it binds, whereas MutLα will cut the same strand if there is a pre-existing nick. Without a pre-existing nick, MutLα has an equal probability of cutting either strand. (A) Model for the generation of expansion intermediates by differential MutL cleavage. There are six ways to cut the double loop-out, depending on which MutL complex cuts first. The triangles represent cut sites with numbers on the triangles indicating the order of cleavage. Three different intermediates will be generated after cleavage. (i) In the case of intermediates generated by cleavage of different strands, gap-filling the two looped-out regions will result in expansion. (ii) When both cuts occur on the same strand, excision or strand-displacement results in the removal of one loop-out. After gap-filling by Polδ the original allele will be restored (no change). (B) The expansion probabilities in cells with different relative levels of MLH3 and PMS2. In cells with sufficient MLH3, PMS2 deficiency will reduce the possibility of cleavages that required PMS2. Thus, the likelihood of MLH3 making both cleavages will increase, resulting in an increase in expansion. More loop-outs will be cleaved by MLH3 in the absence of PMS2, leading to a higher level of expansion. In cells insufficient in MLH3, PMS2 is required to make the first cut in the presence of MLH3 to generate expansion. However, PMS2 deficiency will reduce the possibility of PMS2 making two cleavages, thus increasing the chance of MLH3 making the second cleavage, which results in increased expansion. Insufficient MLH3 to make two cleavages in the absence of PMS2 will result in little or no expansion. The fainter (or absent) graphic reflects its reduced probability.

The fact that a PMS2 expression construct with a point mutation in the nuclease domain is unable to restore expansions suggests that, as we had previously shown for MLH3 (33), PMS2’s nuclease activity is required for its role in promoting expansions. The requirement for both the MLH3 nuclease and the PMS2 nuclease domains suggests that two sets of cleavages are required to generate an expansion. This lends support for a model in which the expansion substrate has two loop-outs as shown in Fig. 7. *In vitro*, MLH3 cleaves the DNA strand opposite any loop-out to which it binds (34). In contrast, on nicked substrates *in vitro*, PMS2 cleaves the nicked strand; while in the absence of a nick, it has an equal probability of cutting either strand (35–37). The simplest interpretation of our data would be consistent with these same cleavage preferences *in vivo*. Strand misalignment during transcription might generate a substrate with a loop-out on each strand. Subsequent cleavage by MutLγ would always result in cuts on opposite strands. These could be processed by exonucleases or by strand-displacement by Polδ to generate a pair of offset gaps located opposite each loop-out. Subsequent gap-filling by Polδ would result in the addition of repeats corresponding to the size of a single loop-out. On the other hand, cleavage by MutLα would have different effects depending on whether MutLγ was also available and which MutL complex cut first. Thus, when MutLγ was available, the loop-outs would sometimes be processed to generate an intermediate with offset gaps on both strands that would result in expansions as illustrated in Fig. 7A-(i). However, at some frequency in the presence of MutLγ, or whenever MutLγ was unavailable, two cuts on the same strand of the loop-out substrate would be generated as illustrated in Fig. 7A-(ii). After processing, an intermediate with a gap on one strand would result, and gap-filling of this intermediate could restore the original allele. Our work in a mESC model of the FXDs and a human iPSC model of GLSD demonstrate an essential role for the 3^rd^ MLH1-binding partner, PMS1, in expansion (12, 24). Given that PMS1 does not have an endonuclease domain but does have an HMG box, a motif associated with many DNA bending proteins, it may be that MutLβ, the PMS1-containing MutL complex, contributes to expansion via a structural role in facilitating the action of MutLγ and MutLα.

One prediction of this model is that there would be an upper limit to PMS2’s ability to drive expansions in cells where PMS2 is required since PMS2 would compete with MLH3 and PMS1 for binding to MLH1 (38) and/or the expansion substrate. This prediction is consistent with fact that while increasing DOX concentrations initially cause a progressive increase in expansions in *Pms2* null mESCs, higher DOX/PMS2 levels do not result in additional expansions or a plateauing as might be expected if some other factor, like the amount of MLH1 or the expansion substrate, was rate-limiting. Instead, high PMS2 levels result in a significant decrease in expansion (Fig. 5), since processing of the expansion substrate by MutLα alone favors expansion-free repair.

While the loss of PMS2 had similar effects at both repeats in many organs, some differences were seen. For example, in cerebellum and liver of *Pms2* null mice, there are more expansions relative to *Pms2*^+/−^ mice for the FXD repeat, but fewer expansions for the HD repeat. This would be consistent with PMS2 playing more of a role driving expansion of the HD repeat in those organs. In the case of liver, this may be related to the more extensive expansions of the HD repeat observed in the liver of *Pms2*^+/+^ animals (Fig. 1 and Fig. 2). More expansions would be consistent with the formation of more of the expansion substrate. This in turn would increase the dependence on PMS2 to process them all. There are other differences between the FXD and HD mice that may have a different underlying explanation. For example, while heterozygosity for *Pms2* results in an increase in expansions of both the FXD and the HD repeat in testes, nullizygosity has different outcomes at each repeat. While it results in contractions of the HD repeat, a small amount of expansion of the FXD repeat is still seen. The difference in the behavior of the two repeats in testes may be related to differences with respect to the major cell type in which the two repeats expand, namely the spermatogonia (SPGs) in the case of *Fmr1* (29) and in haploid gametes for *Htt* (30).

Another small, but potentially important difference between the two repeats, is seen in the striatum, where a population of cells show PMS2-dependent contractions of the HD repeat but not the FXD repeat (Fig. 1 and 2). The reason for this difference is currently under investigation. A better understanding of the mechanism involved in generating these contractions may help identify new therapeutic targets for counteracting somatic expansion.

Despite these differences, our data support the idea that in the case of both the HD and FXD repeats, PMS2 can act both pro-mutagenically to promote expansions and anti-mutagenically to protect against them depending on the cellular context. This reconciles the different observations made in different model systems with the GWAS data from HD patient cohorts and thus lends support to the idea that different REDs share a similar or common expansion mechanism. This has implications for our understanding of the mechanisms controlling repeat instability in this group of disorders. It also increases the confidence that a successful approach for reducing somatic expansions in one of these diseases will be useful to the other diseases in this group.

## Materials and Methods

### Reagents and services

Reagents were from Sigma-Aldrich (St Louis, MO) unless otherwise stated. Primers were from Life Technologies (Grand Island, NY). Capillary electrophoresis of fluorescently labeled PCR products was carried out by the Roy J Carver Biotechnology Center, University of Illinois (Urbana, IL) and Psomagen (Rockville, MD).

### Mouse generation, breeding, and maintenance

Embryos of *Pms2* mutant mice (39) were obtained from The Jackson Laboratory (Bar Harbor, ME; JAX stock #010945) and recovered by NIDDK Laboratory Animal Sciences section (LASS) using standard procedures. The HD mice (zǪ175: B6J.129S1-*Htt*^*tm1Mfc*^/190ChdiJ) (40, 41) were acquired from The Jackson Laboratory (Bar Harbor, ME; JAX stock #027410). The FXD mice (42) have been previously described. *Pms2* mutant mice were crossed to FXD and HD mice to generate animals that were heterozygous for *Pms2*. These mice were then crossed again with FXD or HD mice to generate mice homozygous for the *Pms2* mutation. All mice were on a C57BL/6J background. Mice were maintained in a manner consistent with the Guide for the Care and Use of Laboratory Animals (NIH publications no. 85–23, revised 1996) and in accordance with the guidelines of the NIDDK Animal Care and Use Committee, who approved this research (ASP-K021-LMCB-21).

### Generation of doxycycline-inducible Pms2 constructs

Two plasmids, iPMS2-WT and iPMS2-D696N, were generated to express either WT PMS2 or a nuclease-dead version of PMS2 (D696N) (35) under the control of a doxycycline-inducible promoter. In both of these constructs, an hPGK and an mPGK promoter drive constitutive expression of the doxycycline-responsive TetOn-3G gene and the mClover3 green fluorescent reporter gene (from pKK-TEV-mClover3, Addgene #105795), respectively, as shown in Fig. S2A. The *Pms2* coding sequence was placed downstream of the doxycycline-inducible promoter. The sequence encoding PMS2 corresponds to NCBI Reference Sequence NP_032912.2, with a 1x FLAG epitope sequence inserted immediately after the first codon, and the final codon replaced with an alternate stop codon. In the PMS2-D696N version of the construct, an AAC codon (asparagine) replaces the GAC codon (aspartic acid) at the position corresponding to amino acid 696 of the WT PMS2. These elements are flanked by left and right ROSA homology arms from pROSA26–1 (Addgene #21714) for targeting the construct to endogenous ROSA26 locus. Fragments were combined using standard techniques including Gibson Assembly and NEBuilder HiFi reagents (New England Biolabs, Ipswich, MA). Final construct sequences were confirmed by Sanger sequencing (Psomagen, Inc., Rockville, MD) and whole-plasmid sequencing (Plasmidsaurus, Inc., Louisville, KY).

### Generation and culture of mESCs

The double knock-in mouse ESC (dKI-mESC) carrying both FXD and HD knock-in alleles were derived from embryos obtained by crossing FXD and HD mice using standard procedures and routinely cultured as previously described (43). *Pms2* null alleles were generated in an dKI-mESC line with ~190 FXD repeats and ~228 HD repeats using a CRISPR-Cas9 approach as described previously (24). A *Pms2*^−/−^ dKI-mESC line was transfected with constructs that express either WT PMS2 (iPMS2-WT) or a nuclease-dead version of PMS2 (iPMS2-D696N) under the control of a doxycycline-inducible promoter, described above. These constructs were targeted to the ROSA26 locus of the *Pms2*^−/−^ dKI-mESC lines by co-transfection with a Cas9-expressing plasmid (12), that had been modified to contain gRNAs for the ROSA26 locus. Single-cell-derived lines with stable integration of the transfected construct were identified by expression of a constitutively expressed mClover3 fluorescent reporter protein. Culture media for mESCs was supplemented with DOX at concentrations indicated for various experiments. DOX-induction of the WT and D696N PMS2 was verified both by RT-qPCR and western blotting using standard procedures. For a given DOX concentration the amount of DOX-induced WT PMS2 protein produced was ~2-fold higher than the D696N protein (Fig. S2). However, this does not reflect differences in the protein stability since the amount of PMS2 mRNA produced showed a similar difference (Fig. S2). The reason for this difference is unclear but is frequently seen with this integration strategy and may reflect differences in the number of copies of the expression construct that were integrated.

### DNA isolation

DNA for genotyping was extracted from mouse tails collected at 3-weeks-old, or weaning, using KAPA Mouse Genotyping Kit (KAPA Biosystems, Wilmington, MA). DNA was isolated from a variety of tissues that were collected from 4- and 8-month-old male mice using a Maxwell^*®*^ 16 Mouse Tail DNA Purification kit (Promega, Madison, WI) according to the manufacturer’s instructions. A 5 cm section of the jejunum was collected as the small intestine sample and a 5 cm distal colon sample was collected upstream of the anus as previously described (44). Sperm collection and DNA preparation were as previously described (45). DNA was purified from mESCs as described previously (24).

### Genotyping and analysis of repeat number

Genotyping of *Pms2* was carried out using the KAPA mouse genotyping kit (KAPA Biosystems) according to manufacturer’s instructions with primers JAX-9366 (5’-TTCGGTGACAGATTTGTAAATG-3’) and JAX-9367 (5’-TCACCATAAAAATAGTTTCCCG-3’) used to detect the WT *Pms2* allele and JAX-9366 and JAX-9368 (5’-TTTACGGAGCCCTGGC-3’) to detect the mutant *Pms2* allele. The PCR mix for the *Pms2* allele contained 2 μL template DNA, 1X KAPA2G Fast HotStart Genotyping Mix (KAPA Biosystems, Wilmington, MA), and 0.5 μM each of the primers. The *Pms2* allele PCR conditions were 95°C for 3 minutes; 35 cycles of 95°C for 15 seconds, 60°C for 15 seconds and 72°C for 15 seconds; followed by 72°C for 3 minutes. Genotyping and repeat size analysis of the *Fmr1* and *Htt* alleles was performed using a fluorescent PCR assay with fluorescein amidite (FAM)-labeled primer pairs. The primers FAM-labeled FraxM4 (FAM-5’-CTTGAGGCCCAGCCGCCGTCGGCC-3’) and FraxM5 (5’-CGGGGGGCGTGCGGTAACGGCCCAA-3’) were used for the *Fmr1* allele (42). The PCR mix for *Fmr1* allele contained 3 μL (150 ng) template DNA, 1X KAPA2G Fast HotStart Genotyping Mix, 2.4 M betaine, 2% DMSO, 0.5 μM each of the primers and additional of 125 μM each of dCTP and dGTP. The PCR cycling parameters for the *Fmr1* allele were 95°C for 10 minutes; 35 cycles of 95°C for 30 seconds, 65°C for 30 seconds and 72°C for 90 seconds; followed by 72°C for 10 minutes. The primers FAM-labeled HU3 (FAM-5’-GGCGGCTGAGGAAGCTGAGGA-3’) and Htt-EX1-F1 (5’-GCAACCCTGGAAAAGCTGATGAAGGC-3’) were used for the *Htt* allele. The PCR mix for the *Htt* allele contained 2 μL (100 ng) DNA template, 1x KAPA2G Fast HotStart Genotyping Mix, 1.2 M betaine, 1% DMSO, and 0.5 μM each of the primers. The *Htt* allele was amplified by touchdown PCR using the following parameters: 95°C for 10 minutes; 10 cycles of 95°C for 30 seconds, 72°C with −1°C/cycle for 30 seconds and 72°C for 90 seconds; 28 cycles of 95°C for 30 seconds, 63°C for 30 seconds and 72°C for 90 seconds; followed by 72°C for 10 minutes. The *Fmr1* and *Htt* PCR products were resolved by capillary electrophoresis on an ABI Genetic Analyzer and the resultant fsa files were displayed using a previously described custom R script (46) that is available upon request. The tail sample that was taken at 3-weeks or weaning was used as a proxy-indicator of the original inherited allele size. The expansion index (EI) was calculated in the same way as the somatic instability index (47), but only peaks larger than the original inherited allele were considered, with a cutoff of 10% relative peak height threshold. The repeat number changes were determined by subtracting the number of repeats in the modal allele from the number of repeats in the original inherited allele.

### Statistical analyses

Statistical analyses were performed using GraphPad Prism 10.2. For comparisons of EI or repeat number changes in samples with different genotypes or ages, statistical significance was assessed using the two-way ANOVA with Tukey’s multiple comparisons correction.

## Supplementary Material

Supplement 1

## Data Availability

All data generated or analyzed during this study are included in this published article and its Supplementary Information files.
