# Reconciling the effects of PMS2 in different repeat expansion disease models supports a common therapeutic strategy

**DOI:** 10.1101/2024.08.13.607839

**Published:** 2025-06-04

**Authors:** Diego Antonio Jimenez, Carson J. Miller, Alexandra Walker, Kusala Anupindi, Bruce E. Hayward, Hernan A. Lorenzi, Karen Usdin, Xiaonan Zhao

**Affiliations:** 1 Section on Gene Structure and Disease, Laboratory of Cell and Molecular Biology, National Institute of Diabetes and Digestive and Kidney Diseases, National Institutes of Health, Bethesda, MD 20892; 2 The TriLabs Bioinformatics Group, Laboratory of Biochemistry and Genetics, National Institute of Diabetes and Digestive and Kidney Diseases, National Institutes of Health, Bethesda, MD 20892

**Keywords:** microsatellite instability, MutLα, MutLγ, Huntington’s disease, fragile X-related disorders

## Abstract

Expansion of a disease-specific tandem repeat is responsible for >45 Repeat Expansion Diseases (REDs). The expansion mutation in each of these diseases has different pathological consequences and most are currently incurable. If the underlying mechanism of mutation is shared, a strategy that slows repeat expansion in one RED may be applicable to multiple REDs. However, the fact that PMS2, a component of the MutLα mismatch repair complex, promotes expansion in some models and protects against it in others, suggests that the expansion mechanisms may differ. We show here using mouse models of two REDs caused by different repeats that the seemingly paradoxical effects of PMS2 do not reflect different expansion mechanisms but rather cell-type and dosage effects in different tissues. This differential effect is recapitulated in mouse embryonic stem cells with inducible PMS2 expression: PMS2 promotes expansion at low concentrations, an effect that requires a functional nuclease domain; while at higher concentrations it protects against expansion. The apparent paradoxical behavior of PMS2 can be resolved in a model based on the different *in vitro* cleavage preferences of MutLα and MutLγ, another MutL complex known to be required for expansion*.* Our data thus resolve a longstanding puzzle and suggest a common mechanism responsible for REDs. Our data also provide proof of concept that increasing PMS2 levels suppresses repeat expansion not only in cells where its loss promotes expansion, but also in cells that require it for expansion, supporting its potential as a broadly applicable therapeutic strategy.

## Significance statement

Collectively the Repeat Expansion Diseases (REDs) represent a significant health burden. Since the consequences of the expansion mutation differ across diseases, therapeutic approaches that block the underlying mutation are appealing, particularly if the mechanism is shared. However, the conflicting effects of PMS2 loss in different REDs models challenges this idea. Here we show using two different REDs models that these disparate effects can be reconciled into a single model of repeat expansion, thus increasing confidence that the REDs do all share a common mutational mechanism. Importantly, we show that elevating PMS2 levels suppresses repeat expansion in multiple cellular contexts, including those in which PMS2 is normally required for expansion, demonstrating its potential as a broadly applicable therapeutic strategy for REDs.

## Introduction

Repeat expansion, the increase in the number of repeats in a short tandem repeat (STR) in a disease-specific gene, is the cause of the Repeat Expansion Diseases (REDs), a group of >45 life-limiting neurological or neurodevelopmental disorders (1) with a collective allele frequency of 1 in 283 individuals (2). Converging evidence from studies of genetic modifiers in patients with different REDs implicates components of the Mismatch Repair (MMR) pathway as modifiers of both repeat expansion and disease severity (3–7). This has raised the possibility that targeting some of these factors may be useful therapeutically (8), an appealing idea since these diseases currently have no effective treatment or cure. Furthermore, if indeed these diseases share a common mechanism, a single treatment may be useful for multiple diseases in this group. Some of the same factors identified as modifiers of somatic expansion in human studies have been implicated in expansion in different cell and mouse models of these disorders. For example, MLH3, the binding partner of MLH1 in the MutLγ complex involved in lesion processing in MMR, is required for expansion in multiple disease models (9–16). Taken together these findings suggest that different REDs may expand using the same or very similar mechanisms and that these cell and mouse models are suitable for understanding the expansion process in humans.

However, discordant effects have been seen for some of the MMR factors in different diseases and disease models, casting doubt on the idea that all REDs share a common mechanism or, at least, suggesting that there are important differences between diseases for some important genetic modifiers. For example, PMS2, the MLH1 binding partner in MutLα, another lesion processing complex in MMR, promotes repeat expansion in some models but protects against it in others. Genome wide association studies (GWAS) have shown that PMS2 is a modifier of age at onset and risk of somatic expansion in Huntington’s disease (HD) (17, 18), a CAG-repeat expansion disorder (19). Somatic expansion in HD has been linked to modifier haplotypes that are associated with both earlier and later onset (6, 18). PMS2 has also been shown to protect against expansion in the brain (20, 21) and liver (22) of HD mouse models as well as in the brain of a mouse model of Friedreich’s ataxia (FRDA) (23), a GAA-repeat expansion disorder (24). However, PMS2 was shown to be required for expansion in embryonic stem cells (ESCs) from a mouse model of the Fragile X-related disorders (FXDs) (25), disorders caused by a CGG-repeat expansion in the Fragile X Messenger Ribonucleoprotein 1 (*FMR1*) gene (26). This requirement for PMS2 was also observed in induced pluripotent stem cells (iPSCs) derived from a patient with Glutaminase Deficiency (GLSD) (12), a CAG-repeat expansion disorder (27). Furthermore, in a mouse model of Myotonic Dystrophy Type 1 (DM1), a CTG-repeat expansion disorder (28), loss of PMS2 results in the loss of ~50% of expansions in many organs (29).

To understand these differences, we systematically compared the effects of PMS2 loss in different organs using two REDs mouse models: a mouse model of the FXDs and a mouse model of HD. Using age-matched animals with similar repeat numbers for each model that were either homozygous for wildtype *Pms2* alleles, or heterozygous or homozygous for *Pms2* null alleles, we found that PMS2 had the same apparently paradoxical effects in both models: increasing expansion in some situations and suppressing it in others, an effect that was related to cell type and heterozygosity or homozygosity for the *Pms2* null allele. To better understand this effect, we measured the expansion rate of the FXD and HD repeats in mouse embryonic stem cells (mESC) expressing PMS2 driven by a doxycycline-regulatable promoter. We found that expansion rates for both repeats initially increase and then decrease with increasing PMS2 levels, with PMS2’s ability to promote expansion depending on its nuclease domain. Our findings lend support to the idea that many REDs may in fact share a common expansion mechanism and thus that a common strategy to target components of the MMR pathway to reduce somatic expansion in one RED may be relevant to other diseases in this group. Our data also have interesting implications for the nature of the expansion substrate and for the expansion mechanism itself.

## Results

### PMS2 plays a dual role in somatic expansion in an FXD mouse model.

**Figure 1.**
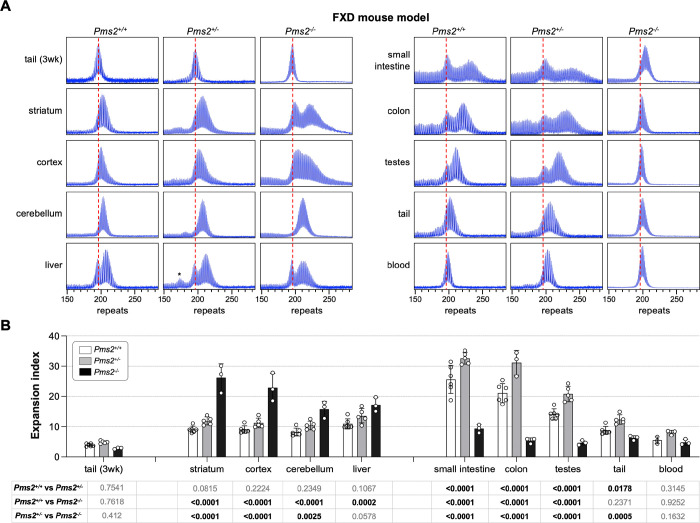
The effect of *Pms2* deficiency on repeat expansion in different tissues of an FXD mouse model. (A) Representative repeat PCR profiles from tail DNA taken at 3 weeks (3 wk) and different organs of 4-month-old *Pms2*^+/+^, *Pms2*^+/−^ and *Pms2*^−/−^ FXD male mice with 196 repeats. The dashed lines represent the sizes of the original inherited alleles as ascertained from the tail DNA taken at 3 weeks. (B) Comparison of the expansion index (EI) in the indicated organs of 4-month-old *Pms2*^+/+^, *Pms2*^+/−^ and *Pms2*^−/−^ FXD mice with an average of 194 repeats in the original allele. The colon data represent the average of 6 *Pms2*^+/+^, 3 *Pms2*^+/−^ and 3 *Pms2*^−/−^ mice with 185–210 repeats. The blood data represent the average of 3 *Pms2*^+/+^, 3 *Pms2*^+/−^ and 3 *Pms2*^−/−^ mice in the same repeat range. The data from other organs represents the average of 6 *Pms2*^+/+^, 5 *Pms2*^+/−^ and 3 *Pms2*^−/−^ mice in the same repeat range. The error bars indicate the standard deviations of the mean. Each dot represents one animal. In each organ, the EIs for different genotypes were compared using a two-way ANOVA with correction for multiple testing as described in the Materials and Methods. The adjusted P-values are listed in the table below. The asterisks in the *Pms2*^+/−^ liver sample indicates a contracted allele that is also present in other organs and not a specific contraction caused by PMS2 deficiency.

To examine the role of PMS2 in repeat expansion in an FXD mouse model, we crossed FXD mice to mice with a null mutation in *Pms2*. We then examined the expansion profiles in animals matched for age and repeat number. As can be seen in Fig. 1, heterozygosity for *Pms2* results in an increase in expansion in most, if not all, expansion-prone tissues including striatum, liver and small intestine. This would be consistent with the interpretation that PMS2 normally protects against expansion. In *Pms2*^−/−^ mice, a further increase in expansion was seen in some tissues, including the striatum, cortex, cerebellum, and liver, that would also be consistent with this interpretation. However, in other organs, including small intestine, colon and blood, fewer expansions were seen in *Pms2* null mice than were seen in heterozygous mice. Thus, in the very same animals, PMS2 can both promote and prevent expansion depending on cell type and the levels of PMS2 expressed.

### PMS2 plays a similar dual role in somatic expansion in an HD mouse model.

**Figure 2.**
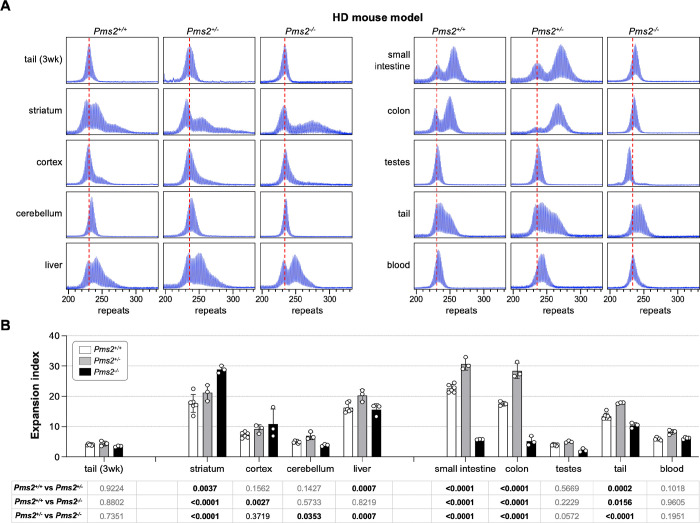
The effect of *Pms2* deficiency on repeat expansion in different tissues from an HD mouse model. (A) Representative repeat PCR profiles from tail DNA taken at 3 weeks (3 wk) and different organs of 4-month-old *Pms2*^+/+^, *Pms2*^+/−^ and *Pms2*^−/−^ HD male mice with ~230 repeats. The dashed lines represent the sizes of the original inherited alleles as ascertained from the tail DNA taken at 3 weeks. (B) Comparison of the expansion index (EI) in the indicated organs of 4-month-old *Pms2*^+/+^, *Pms2*^+/−^ and *Pms2*^−/−^ HD mice with an average of 234 repeats in the original allele. The colon data represent the average of 3 *Pms2*^+/+^, 3 *Pms2*^+/−^ and 3 *Pms2*^−/−^ mice with 226–239 repeats. The data from other organs represents the average of 6 *Pms2*^+/+^, 3 *Pms2*^+/−^ and 3 *Pms2*^−/−^ mice in the same repeat range. The error bars indicate the standard deviations of the mean. Each dot represents one animal. In each organ, the EIs for different genotypes were compared using a two-way ANOVA with correction for multiple testing as described in the Materials and Methods. The adjusted P-values are listed in the table below.

To assess the role of PMS2 in repeat expansion in an HD mouse model, we crossed HD mice to the same *Pms2* null mice and again assessed repeat instability in animals matched for age and repeat number. In the HD mouse model, as in the FXD mouse model, heterozygosity for *Pms2* results in an increase in expansions in most expansion-prone tissues (Fig. 2). Furthermore, striatum, which showed more extensive expansions in FXD mice nullizygous for *Pms2*, also showed a similar increase in the extent of expansion in the HD mice. Interestingly, in animals WT for *Pms2*, a population of cells in the striatum had alleles that were smaller than the allele seen in the tail DNA taken at weaning. The proportion of contracted alleles decreased with decreasing *Pms2* gene dosage suggesting that the contractions are PMS2-dependent.

However, as was seen in the FXD mice, in other organs loss of all PMS2 resulted in a decrease rather than in increase in expansions. In contrast to the FXD mouse, this was also the case in liver and cerebellum. Furthermore, the increase of expansion in the striatum and cortex of the *Pms2* null HD mice were smaller than in the *Pms2* null FXD mice, with the difference between heterozygous and nullizygous mice not reaching statistical significance for the cortex. Another difference between the mouse models was seen in the testes. While expansions in the FXD testes of *Pms2*^+/+^ mice were extensive, they were relatively modest in the testes of *Pms2*^+/+^ HD mice. This is consistent with previous work showing that FXD repeat expansions in mouse testes occurs primarily in the spermatogonial cells (SPGs) (30), whereas HD expansions in both mice and humans occur in the spermatozoa (31–33). Since the spermatogonia are the reservoir of replicating cells from which mature gametes are derived, while spermatozoa are a short-lived cell type, successive expansions can accumulate over time in SPGs but not in spermatozoa. This may explain why the repeat number seen in mature sperm increases significantly over time in FXD mice but not in HD mice. Despite this difference, as in other expansion-prone tissues, FXD and HD repeats in testes expanded faster in *Pms2*^+/−^ mice than in *Pms2*^+/+^ mice. However, unlike the FXD repeats in *Pms2*^−/−^ testes where the modal allele was slightly larger than the modal allele in tail taken at weaning, the HD repeat number in *Pms2*^−/−^ testes was smaller than it was in the initial inherited allele, consistent with contractions.

**Figure 3.**
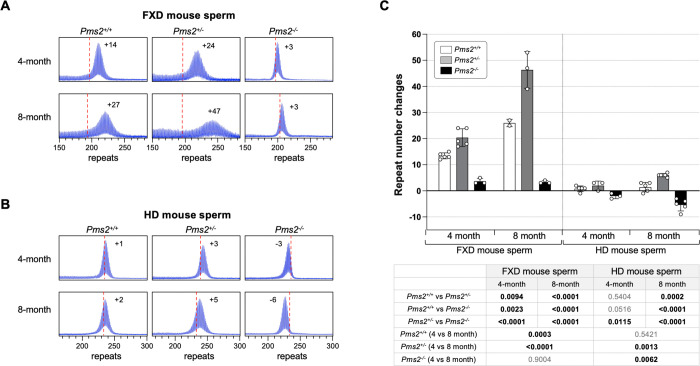
The effect of *Pms2* deficiency on repeat instability in sperm from HD and FXD mouse models. (A) Representative repeat PCR profiles from 4- and 8-month-old *Pms2*^+/+^, *Pms2*^+/−^ and *Pms2*^−/−^ HD male mice with ~230 repeats. The number associated with each profile indicates the change in repeat number relative to the original inherited allele. The dashed lines represent the sizes of the original inherited alleles as ascertained from the tail DNA taken at 3 weeks. (B) Representative repeat PCR profiles from 4- and 8-month-old *Pms2*^+/+^, *Pms2*^+/−^ and *Pms2*^−/−^ FXD male mice with ~197 repeats. The number associated with each profile indicates the change in repeat number relative to the original inherited allele. The dashed lines represent the sizes of the original inherited alleles as ascertained from the tail DNA taken at 3 weeks. (C) Comparison of the repeat number changes in the sperm of 4- and 8-month-old *Pms2*^+/+^, *Pms2*^+/−^ and *Pms2*^−/−^ mice. The 4-month-old HD mice data represents the average of 5 *Pms2*^+/+^, 3 *Pms2*^+/−^ and 3 *Pms2*^−/−^ mice with 229–239 repeats (average of 234 repeats) in the original allele. The 8-month-old HD mice data represents the average of 5 *Pms2*^+/+^, 6 *Pms2*^+/−^ and 5 *Pms2*^−/−^ mice with 219–235 repeats (average of 225 repeats) in the original allele. The 4-month-old FXD mice data represents the average of 5 *Pms2*^+/+^, 5 *Pms2*^+/−^ and 3 *Pms2*^−/−^ mice with 185–210 repeats (average of 193 repeats) in the original allele. The 8-month-old FXD mice data represents the average of 2 *Pms2*^+/+^, 3 *Pms2*^+/−^ and 3 *Pms2*^−/−^ mice with 197–224 repeats (average of 209 repeats) in the original allele. The error bars indicate the standard deviations of the mean. Each dot represents one animal. In each mouse model, the repeat number changes with different genotype and age were compared using a two-way ANOVA with correction for multiple testing as described in the Materials and Methods. The adjusted P-values are listed in the table below.

To study this phenomenon in more detail, we compared the change in the modal repeat length in the sperm of *Pms2*^+/+^, *Pms2*^+/−^ and *Pms2*^−/−^ mice at 4 months and 8 months of age. As can be seen in Fig. 3, while the repeat number remained stable in the sperm of FXD mice lacking PMS2, it decreased with age in the sperm of HD mice. Thus, contractions occurring in the HD repeats in the testes are different from the contractions observed for the HD repeats in the striatum in that they are not PMS2-dependent. Rather they resemble the typical microsatellite instability associated with an MMR deficiency as we previously observed in stem cell models of different REDs (7, 25).

### The differential effects of PMS2 are related to gene dosage and are dependent on the PMS2 nuclease domain.

**Figure 4.**
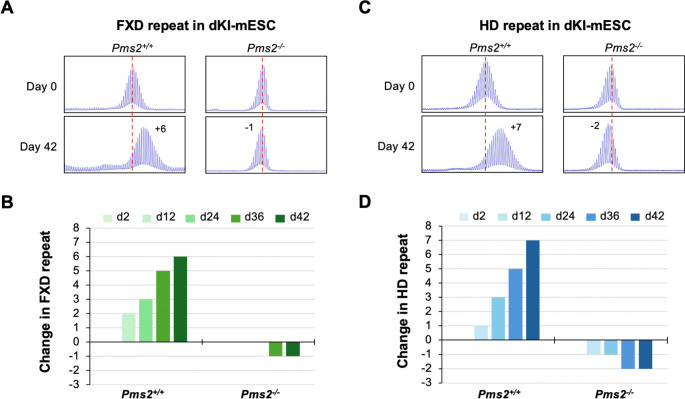
Effect of *Pms2* deficiency on expansion of FXD and HD repeats in a double knock-in mESC model. (A, C) Repeat PCR profiles of FXD (A) and HD (C) repeats in *Pms2*^+/+^ and *Pms2*^−/−^ dKI-mESCs carrying both FXD and HD repeats. The numbers in the day 42 profiles indicate the change in repeat number. The red dotted line indicates the starting allele. (B, D) Changes in FXD (B) and HD (D) repeats number at the indicated days (d) in culture.

These results suggest that whether PMS2 protects against or promotes expansion is related to the relative amounts of PMS2 and MLH3. To address this issue further we generated double knock-in (dKI) mESCs carrying ~180 FXD repeats and ~215 HD repeats. These lines showed similar rates of expansion as single knock-in cell lines (Fig. S1). We then used CRISPR-Cas9 to knock out *Pms2* in a dKI-mESC line. No expansion of either repeat was seen in these lines (Fig. 4). Thus, PMS2 is required for expansions of both repeats in this model system, consistent with our previous demonstration for the FXD repeat (25) as well as the CAG repeat responsible for GLSD (7). As we had previously observed, complete loss of PMS2 resulted in small decreases in repeat number for both repeats. This is consistent with the idea that MLH3 levels in this cell type are too low to support expansion without a contribution from PMS2.

**Figure 5.**
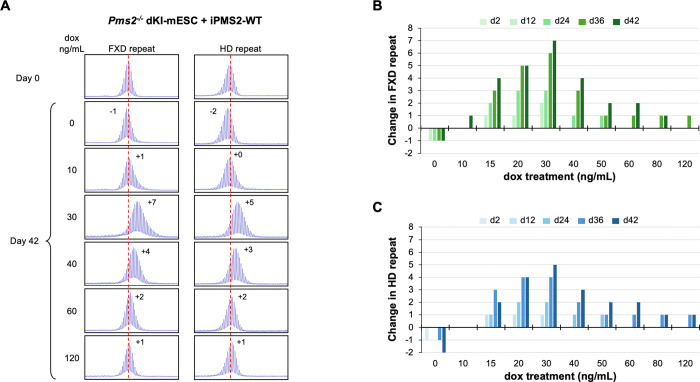
Effect of doxycycline-induced WT PMS2 expression on expansion of FXD and HD repeats in a double knock-in mESC model. (A) Repeat PCR profiles of FXD (left) and HD (right) repeats in *Pms2*^−/−^ dKI-mESCs expressing DOX-induced WT PMS2 (iPMS2-WT) at different concentrations of DOX after 42 days in culture. The number associated with each profile indicates the change in repeat number. The red dotted line indicates the starting allele. DOX concentrations producing similar levels of both DOX-induced WT and D696N versions of the DOX-induced PMS2 protein were used. (B, C) Changes in FXD (B) and HD (C) repeat number over time in culture in *Pms2*^−/−^ dKI-mESCs expressing DOX-induced WT PMS2 at different concentrations of DOX. Days in culture (d) indicated above graphs.

**Figure 6.**
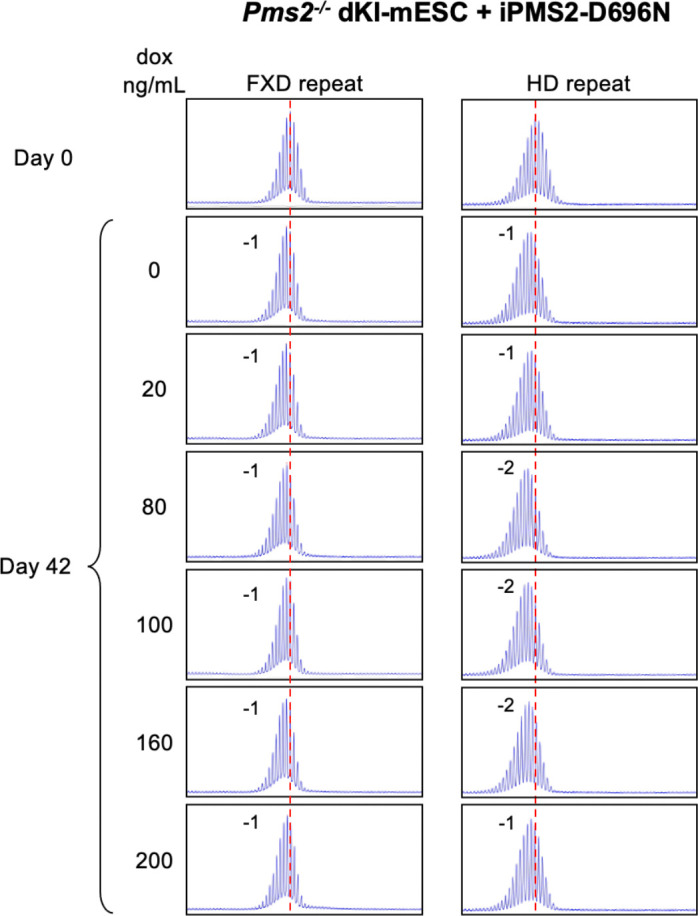
Effect of doxycycline-induced PMS2-D6G6N expression on expansion of FXD and HD repeats in a double knock-in mESC. (A) Repeat PCR profiles of FXD (left) and HD (right) repeats in *Pms2*^−/−^ dKI-mESCs expressing DOX-induced PMS2 D696N (iPMS2-D696N) at different concentrations of DOX after 42 days in culture. The number associated with each profile indicates the change in repeat number. The red dotted line indicates the starting allele. DOX concentrations producing similar levels of both DOX-induced WT and D696N versions of the DOX-induced PMS2 protein were used.

We then integrated a doxycycline (DOX)-inducible WT PMS2-expressing construct into a *Pms2*^−/−^ dKI-mESC line and monitored the stability of both the FXD and the HD repeats over time in different concentrations of DOX. Without DOX treatment, small contractions like those seen in *Pms2*^−/−^ lines were also seen (Fig. 5). As the concentration of DOX was increased, so a progressive increase in repeat expansions was seen for both repeats reaching a maximum at 30 ng/mL. The extent of expansions at this DOX concentration was comparable to the extent of expansions seen in *Pms2*^+/+^ cells (Fig. 4). Higher concentrations of DOX resulted in a progressive decrease in expansions of both repeats. Thus, both too little PMS2 and too much can result in decreased expansion. Notably, in contrast to the contractions seen when PMS2 is lost, a low level of expansion was still seen after extended growth at the highest DOX concentrations when PMS2 was overexpressed. We repeated this experiment with a construct expressing similar levels of PMS2 containing a D696N mutation in the nuclease domain (Fig. S2). As can be seen in Fig. 6, only small contractions, like those seen in *Pms2*^−/−^ line, were seen for both repeats at all DOX concentrations tested. Thus, an intact nuclease domain is required for PMS2’s promotion of expansion.

## Discussion

We show here that mice heterozygous for *Pms2* show more expansion of the FXD and HD repeats in expansion-prone tissue than *Pms2*^+/+^ mice (Fig. 1 and Fig. 2). This is consistent with PMS2 playing a role in preventing expansion of both the FXD and the HD repeats. However, in nullizygous animals, while some organs showed a further increase in expansions of both repeats consistent with a protective role for PMS2, other organs showed a significant decrease relative to heterozygous and WT animals. Thus, in some organs or cell types, PMS2 promotes expansions *i.e.*, it can act pro-mutagenically. This idea is substantiated by our demonstration in that loss of PMS2 eliminates expansions of both the FXD and the HD repeats in mESCs (Fig. 4). Then, when levels of PMS2 are systematically increased in a *Pms2*^−/−^ cell line, expansion rates rise, peaking at expansion rates similar to those seen in WT cells for both repeats. However, when the PMS2 levels are increased even further, expansion rates begin to decline (Fig. 5). Therefore, the different effects of PMS2 reported in different model systems of different REDs likely do not reflect fundamentally different mechanisms of instability in these diseases, but rather the effect of different levels of PMS2 in different cellular contexts.

**Figure 7.**
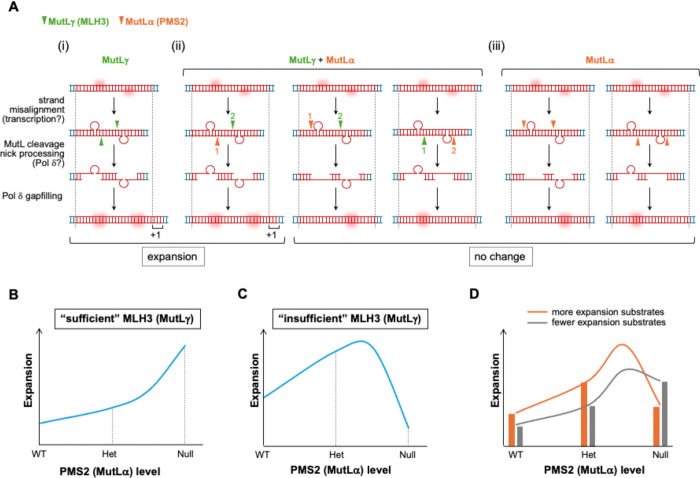
Model for the differential effects of a PMS2 deficiency on the probability of repeat expansion. A double loop-out structure can form in the region of repeats when the DNA is transiently unpaired. Repeats are shown in red, and shaded areas indicate the repeats that correspond to the loop-outs. Depending on the relative abundance of different MMR proteins and their relative binding affinities, each loop-out is bound by either MutLγ or MutLα, and then either the same strand or the opposite strand of the loop-out will be cleaved by the MutL complexes. MutLγ always cuts the opposite strand of the loop-out it binds, whereas MutLα will cut the same strand if there is a pre-existing nick. Without a pre-existing nick, MutLα has an equal probability of cutting either strand. (A) Model for the generation of expansion intermediates by differential MutL cleavage. There are six ways to cut the double loop-out, depending on which MutL complex cuts first. The triangles represent cut sites with numbers on the triangles indicating the order of cleavage. Three different intermediates will be generated after cleavage. (i) In the case of intermediates generated by cleavage of different strands, gap-filling the two looped-out regions will result in expansion. (ii) When both cuts occur on the same strand, excision or strand-displacement results in the removal of one loop-out. After gap-filling by Polδ the original allele will be restored (no change). (B)-(D) Simulation of the expansion probabilities based on the amount of the expansion substrate and the relative levels of MLH3 and PMS2. (B) In cells with sufficient MLH3 to process all expansion substrates, MutLα competes with MutLγ for substrate processing. Since MutLα cleavage does not always produce expansions, a decrease in PMS2 levels always leads to an increase in the total number of possible expansions. Simulation curve generated using 100 loop-outs, 100 molecules of MutLγ, and decreasing levels of MutLα at 200, 100, 50, and 0. (C) In cells that lack sufficient MutLγ to process all expansion substrates without a contribution from MutLα, decreasing levels of MutLα might initially cause an increase in expansions as the ratio of MutLγ to MutLα comes to favor processing of the substrate by both MutL complexes. However, expansions would begin to decrease when PMS2 levels dropped beyond a certain point since levels of MutLγ are too low to result in processing of both loop-outs. Simulation curve generated using 100 loop-outs, 10 molecules of MutLγ, and decreasing levels of MutLα at 200, 100, 50, and 0. (D) Simulation of repeat-specific expansion. Repeats capable of forming more expansion substrates (80 loop-outs, orange line) or fewer substrates (50 loop-outs, gray line) were modeled using 20 molecules of MutLγ, and decreasing levels of MutLα at 200, 100, 50, and 0. Differences in the amount of expansion substrate are sufficient to explain the distinct expansion patterns observed in liver and cerebellum of FXD and HD mice. Modeling assumptions and calculation details are described in the Materials and Methods.

The fact that a PMS2 expression construct with a point mutation in the nuclease domain is unable to restore expansions (Fig. 6) suggests that PMS2’s nuclease activity is required for its role in promoting expansions as we had previously shown for MLH3 (34). The requirement for both the MLH3 and the PMS2 nuclease domains suggests that two sets of cleavages are required to generate an expansion. This provides support for a model in which the expansion substrate has two loop-outs (29), each of which could be processed either by MutLγ or MutLα. *In vitro*, MLH3 cleaves the DNA strand opposite any loop-out to which it binds (35). In contrast, on nicked substrates *in vitro*, PMS2 cleaves the nicked strand; while in the absence of a nick, it has an equal probability of cutting either strand (36–38). The simplest interpretation of our data would be consistent with these same cleavage preferences occurring *in vivo*. Strand misalignment during transcription might generate a substrate with a loop-out on each strand. Subsequent cleavage by MutLγ would always result in cuts on opposite strands. These could be processed by exonucleases or by strand-displacement by Polδ to generate a pair of offset gaps located opposite each loop-out. Subsequent gap-filling by Polδ would result in the addition of repeats to each strand that corresponded to the size of a single loop-out as illustrated in Fig. 7A(i). On the other hand, when MutLα is involved, the outcome would depend on whether generation of a nick at one loop-out affects cleavage at the second loop-out, *i.e.,* if loop processing was coordinated, as it might be if the two loop-outs were situated relatively close together. If it was, then cleavage of second loop-out by MutLα would always occur on the same strand as the first as illustrated in Fig. 7A–(ii) and (iii). This would generate an intermediate with a gap on one strand and gap-filling of this intermediate could restore the original allele. However, if the two loops were instead processed independently, then MutLα processing of both loop-outs could generate a mixture of expansions, contractions and unchanged alleles (Fig. S3A). Mathematical modeling suggests that such processing would result in alleles with a normal allele size distribution that broadens over time (Fig. S3B). This broadening arises from the stochastic nature of these events that results in increased repeat heterogeneity in the population. Such heterogeneity would be evidenced by a progressive increase in the standard deviation of the allele profile as illustrated in Fig. S3B. However, in *Mlh3* null FXD mice, no difference was seen in the CGG-repeat PCR profiles of DNA from tail taken at weaning or at 12 months of age (Fig. S3C). This suggests that a combination of expansions and contractions did not occur, thus favoring the coordinated loop processing model. However, whether the loop-outs are processed independently or in a coordinated fashion may be related to factors including the repeat number, with independent processing perhaps predominating as the repeat number increases.

In either variant of the model, MutLγ processing of both loop-outs would always result in expansions, while MutLα processing of one or both loop-outs would not necessarily do so. Thus, in cells where MutLγ is relatively abundant, reducing the amount of PMS2 increases the likelihood that MutLγ will process the substrate. This would result in a higher expansion rate since competition between MutLγ and MutLα for substrate processing would be reduced as illustrated in the mathematical simulations shown in Fig. 7B. In contrast, when MLH3 is limited or the number of substrates was relatively high, MutLγ alone would be insufficient to process them all. In this case, the absence of PMS2 results in a reduction in expansion as shown in the simulation in Fig. 7C.

One prediction of these models is that there would be an upper limit to PMS2’s ability to drive expansions in cells where PMS2 is required since PMS2 would compete with MLH3 and PMS1, the two other MLH1-binding partners that are also implicated in expansion (12, 25). This prediction is consistent with fact that while increasing DOX concentrations initially cause a progressive increase in expansions in *Pms2* null mESCs, higher DOX/PMS2 levels do not result in additional expansions or a plateauing as might be expected if some other factor, like the amount of MLH1 or the expansion substrate, was rate-limiting. Instead, high PMS2 levels result in a significant decrease in expansion (Fig. 5), consistent with a requirement of MutLγ and/or MutLβ for MutLα’s effect on expansion.

While the loss of PMS2 had similar effects at both repeats in many organs, some differences were seen. For example, in cerebellum and liver of *Pms2* null mice, FXD mice show more expansions than heterozygous mice, while HD mice show fewer. In the case of liver where expansion of both repeats is limited to hepatocytes (39, 40) and thus where the level of PMS2 and MLH3 available to process both repeats would be the same, slightly more expansions of the HD repeat are seen in *Pms2*^+/+^ animals (Fig. 1 and Fig. 2). This could reflect the formation of more of the expansion substrate, which would in turn increase the dependence on PMS2 as is seen in the mathematic simulations of the expansion probabilities shown in Fig. 7D. However, the steady state levels of *Htt* transcripts in hepatocytes is not higher than that of *Fmr1* (Fig. S4). Whether *Htt* has a higher transcription rate than *Fmr1* in hepatocytes remains an open question.

In other cases, the difference between the behavior of the two repeats in a particular organ may be related to differences in the cell types that express the repeat-associated gene. For example, in the testes, *Pms2* nullizygosity results in contractions of the HD repeat while a small amount of expansion of the FXD repeat is still seen. Expansion of the FXD repeat occurs primarily in the spermatogonia (SPGs) (30), where *Fmr1* expression is highest, while most expansion of the HD repeat occurs in spermatids (31) when *Htt* expression is higher (Fig. S4). Proteomics studies show that the level of many MMR components decline as spermatogenesis proceeds (41). Thus, spermatogonia may have “sufficient” MLH3 to process any expansion substrates generated by transcription, while spermatids may have levels that are too low to do so. As a result, in the absence of PMS2, substrate processing in spermatids may be more likely to result in the contractions typical of an MMR-deficiency. This may be analogous to the situation in mESCs, although the absolute levels of the MMR factors and expansion substrates likely differ. Contractions of the FXD repeat in spermatids might not only be less likely to occur since transcript levels are lower, but any that do occur might be masked by the accumulation of a low level of MutLγ-driven expansions occurring in the replicating spermatogonia. Another small, but potentially important, difference between the two repeats is seen in the striatum, where a population of cells show PMS2-dependent contractions of the HD repeat but not the FXD repeat (Fig. 1 and 2). The reason for this difference and whether contractions in HD sperm and striatum are related is currently under investigation. A better understanding of the mechanism involved in generating these contractions may help identify new therapeutic targets for counteracting somatic expansion.

Despite these differences, our data support the idea that in the case of both the HD and FXD repeats, PMS2 can act both pro-mutagenically to promote expansions and anti-mutagenically to protect against them depending on the cell type and gene dosage. This reconciles the different observations made in different model systems with the GWAS data from HD patient cohorts and thus lends support to the idea that different REDs share a similar or common expansion mechanism. This has implications for our understanding of the mechanisms controlling repeat instability in this group of disorders. It also increases the confidence that a successful approach for reducing somatic expansions in one of these diseases will be useful to the other diseases in this group. In particular, our demonstration that relatively modest increases in PMS2 significantly reduce expansions in our mESC model raises the possibility that increasing PMS2 may be a therapeutically useful approach for this whole group of diseases, regardless of whether PMS2 loss increases or decreases expansion. While all strategies that affect the relative levels of MMR proteins come with some risk, there may be ways to mitigate this using co-expression of MLH1 (42) or by making use of naturally occurring (43) or engineered PMS2 variants with higher DNA repair proficiency. One major therapeutic approach already being tested involves reducing the levels of other MMR factors such as MLH3. Our demonstration that the amount of expansion is exquisitely sensitive to the ratio of MLH3 and PMS2 may make some combination of MLH3-reduction and PMS2 enhancement, a strategy worth exploring.

## Materials and Methods

### Reagents and services

Reagents were from Sigma-Aldrich (St Louis, MO) unless otherwise stated. Primers were from Life Technologies (Grand Island, NY). Capillary electrophoresis of fluorescently labeled PCR products was carried out by the Roy J Carver Biotechnology Center, University of Illinois (Urbana, IL) and Psomagen (Rockville, MD).

### Mouse generation, breeding, and maintenance

Embryos of *Pms2* mutant mice (44) were obtained from The Jackson Laboratory (Bar Harbor, ME; JAX stock #010945) and recovered by NIDDK Laboratory Animal Sciences section (LASS) using standard procedures. The HD mice (zǪ175: B6J.129S1-*Htt*^*tm1Mfc*^/190ChdiJ) (45, 46) were acquired from The Jackson Laboratory (Bar Harbor, ME; JAX stock #027410). The FXD mice (47) have been previously described. *Pms2* mutant mice were crossed to FXD and HD mice to generate animals that were heterozygous for *Pms2*. These mice were then crossed again with FXD or HD mice to generate mice homozygous for the *Pms2* mutation. All mice were on a C57BL/6J background. Mice were maintained in a manner consistent with the Guide for the Care and Use of Laboratory Animals (NIH publications no. 85–23, revised 1996) and in accordance with the guidelines of the NIDDK Animal Care and Use Committee, who approved this research (ASP-K021-LMCB-21).

### Generation of doxycycline-inducible Pms2 constructs

Two plasmids, iPMS2-WT and iPMS2-D696N, were generated to express either WT PMS2 or a nuclease-dead version of PMS2 (D696N) (36) under the control of a doxycycline-inducible promoter. In both of these constructs, an hPGK and an mPGK promoter drive constitutive expression of the doxycycline-responsive TetOn-3G gene and the mClover3 green fluorescent reporter gene (from pKK-TEV-mClover3, Addgene #105795), respectively, as shown in Fig. S2A. The *Pms2* coding sequence was placed downstream of the doxycycline-inducible promoter. The sequence encoding PMS2 corresponds to NCBI Reference Sequence NP_032912.2, with a 1x FLAG epitope sequence inserted immediately after the first codon, and the final codon replaced with an alternate stop codon. In the PMS2-D696N version of the construct, an AAC codon (asparagine) replaces the GAC codon (aspartic acid) at the position corresponding to amino acid 696 of the WT PMS2. These elements are flanked by left and right ROSA homology arms from pROSA26–1 (Addgene #21714) for targeting the construct to endogenous ROSA26 locus. Fragments were combined using standard techniques including Gibson Assembly and NEBuilder HiFi reagents (New England Biolabs, Ipswich, MA). Final construct sequences were confirmed by Sanger sequencing (Psomagen, Inc., Rockville, MD) and whole-plasmid sequencing (Plasmidsaurus, Inc., Louisville, KY).

### Generation and culture of mESCs

The double knock-in mouse ESC (dKI-mESC) carrying both FXD and HD knock-in alleles were derived from embryos obtained by crossing FXD and HD mice using standard procedures and routinely cultured as previously described (48). *Pms2* null alleles were generated in an dKI-mESC line with ~190 FXD repeats and ~228 HD repeats using a CRISPR-Cas9 approach as described previously (25). A *Pms2*^−/−^ dKI-mESC line was transfected with constructs that express either WT PMS2 (iPMS2-WT) or a nuclease-dead version of PMS2 (iPMS2-D696N) under the control of a doxycycline-inducible promoter, described above. These constructs were targeted to the ROSA26 locus of the *Pms2*^−/−^ dKI-mESC lines by co-transfection with a Cas9-expressing plasmid (12), that had been modified to contain gRNAs for the ROSA26 locus. Single-cell-derived lines with stable integration of the transfected construct were identified by expression of a constitutively expressed mClover3 fluorescent reporter protein. Culture media for mESCs was supplemented with DOX at concentrations indicated for various experiments. DOX-induction of the WT and D696N PMS2 was verified both by RT-qPCR and western blotting using standard procedures. For a given DOX concentration, the amount of DOX-induced WT PMS2 protein produced was ~2-fold higher than the D696N protein (Fig. S2). However, this does not reflect differences in the protein stability since the amount of PMS2 mRNA produced showed a similar difference (Fig. S2). The reason for this difference is unclear but is frequently seen with this integration strategy and may reflect differences in the number of copies of the expression construct that were integrated.

### DNA isolation

DNA for genotyping was extracted from mouse tails collected at 3-weeks-old, or weaning, using KAPA Mouse Genotyping Kit (KAPA Biosystems, Wilmington, MA). DNA was isolated from a variety of tissues that were collected from 4- and 8-month-old male mice using a Maxwell^®^ 16 Mouse Tail DNA Purification kit (Promega, Madison, WI) according to the manufacturer’s instructions. A 5 cm section of the jejunum was collected as the small intestine sample and a 5 cm distal colon sample was collected upstream of the anus as previously described (49). Sperm collection and DNA preparation were as previously described (50). DNA was purified from mESCs as described previously (25).

### Genotyping and analysis of repeat number

Genotyping of *Pms2* was carried out using the KAPA mouse genotyping kit (KAPA Biosystems) according to manufacturer’s instructions with primers JAX-9366 (5’-TTCGGTGACAGATTTGTAAATG-3’) and JAX-9367 (5’-TCACCATAAAAATAGTTTCCCG-3’) used to detect the WT *Pms2* allele and JAX-9366 and JAX-9368 (5’-TTTACGGAGCCCTGGC-3’) to detect the mutant *Pms2* allele. The PCR mix for the *Pms2* allele contained 2 μL template DNA, 1X KAPA2G Fast HotStart Genotyping Mix (KAPA Biosystems, Wilmington, MA), and 0.5 μM each of the primers. The *Pms2* allele PCR conditions were 95°C for 3 minutes; 35 cycles of 95°C for 15 seconds, 60°C for 15 seconds and 72°C for 15 seconds; followed by 72°C for 3 minutes. Genotyping and repeat size analysis of the *Fmr1* and *Htt* alleles was performed using a fluorescent PCR assay with fluorescein amidite (FAM)-labeled primer pairs. The primers FAM-labeled FraxM4 (FAM-5’-CTTGAGGCCCAGCCGCCGTCGGCC-3’) and FraxM5 (5’-CGGGGGGCGTGCGGTAACGGCCCAA-3’) were used for the *Fmr1* allele (47). The PCR mix for *Fmr1* allele contained 3 μL (150 ng) template DNA, 1X KAPA2G Fast HotStart Genotyping Mix, 2.4 M betaine, 2% DMSO, 0.5 μM each of the primers and additional of 125 μM each of dCTP and dGTP. The PCR cycling parameters for the *Fmr1* allele were 95°C for 10 minutes; 35 cycles of 95°C for 30 seconds, 65°C for 30 seconds and 72°C for 90 seconds; followed by 72°C for 10 minutes. The primers FAM-labeled HU3 (FAM-5’-GGCGGCTGAGGAAGCTGAGGA-3’) and Htt-EX1-F1 (5’-GCAACCCTGGAAAAGCTGATGAAGGC-3’) were used for the *Htt* allele. The PCR mix for the *Htt* allele contained 2 μL (100 ng) DNA template, 1x KAPA2G Fast HotStart Genotyping Mix, 1.2 M betaine, 1% DMSO, and 0.5 μM each of the primers. The *Htt* allele was amplified by touchdown PCR using the following parameters: 95°C for 10 minutes; 10 cycles of 95°C for 30 seconds, 72°C with −1°C/cycle for 30 seconds and 72°C for 90 seconds; 28 cycles of 95°C for 30 seconds, 63°C for 30 seconds and 72°C for 90 seconds; followed by 72°C for 10 minutes. The *Fmr1* and *Htt* PCR products were resolved by capillary electrophoresis on an ABI Genetic Analyzer and the resultant fsa files were displayed using a previously described custom R script (51) that is available upon request. The tail sample that was taken at 3-weeks or weaning was used as a proxy-indicator of the original inherited allele size. The expansion index (EI) was calculated in the same way as the somatic instability index (52), but only peaks larger than the original inherited allele were considered, with a cutoff of 10% relative peak height threshold. The repeat number changes were determined by subtracting the number of repeats in the modal allele from the number of repeats in the original inherited allele.

### Statistical analyses

Statistical analyses were performed using GraphPad Prism 10.2. For comparisons of EI or repeat number changes in samples with different genotypes or ages, statistical significance was assessed using the two-way ANOVA with Tukey’s multiple comparisons correction.

### Mathematical modeling of MutL protein-mediated loop-out processing

A deterministic model was developed to simulate how MLH3 and PMS2 influence the mutational outcome of coordinated loop-out processing. Each simulation considers a defined number of loop-out pairs or expansion substrates in the population. Each substrate is assigned randomly to be processed by either MutLγ or Mutα, with binding probabilities determined by the relative abundance of each protein. For simplicity, equal binding affinities for MutLγ and MutLα were assumed. Once bound, the processing outcome is determined based on six cleavage patterns: two that lead to expansion and four that do not, consistent with the model shown in Fig. 7. The total mutation rate is calculated by summing mutation-causing cleavage events across all loop-outs. All calculations were performed in Excel, and the original spreadsheet is available upon request.

## Supplementary Material

Supplement 1

## Data Availability

All data generated or analyzed during this study are included in this published article and its Supplementary Information files.
