# Supplementary material for: Reconciling the effects of PMS2 in different repeat expansion disease models supports a common therapeutic strategy
Source: bioRxiv. 2025 Jun 4:2024.08.13.607839. Originally published 2024 Aug 13. Preprint. [Version 3] doi: 10.1101/2024.08.13.607839 (PMC11343130; doi:10.1101/2024.08.13.607839)
Supplement: Supplement 1 [file media-1.pdf]

Reconciling the effects of PMS2 in different repeat expansion disease models supports a common therapeutic strategy

Diego Antonio Jimenez[1,a,#], Carson J. Miller[1,#], Alexandra Walker[1,#], Kusala Anupindi[1,b], Bruce E. Hayward[1], Hernan A. Lorenzi[2], Karen Usdin[1,*], and Xiaonan Zhao[1,*]

[1] Section on Gene Structure and Disease, Laboratory of Cell and Molecular Biology, National Institute of Diabetes and Digestive and Kidney Diseases, National Institutes of Health, Bethesda, MD 20892

[2] The TriLabs Bioinformatics Group, Laboratory of Biochemistry and Genetics, National Institute of Diabetes and Digestive and Kidney Diseases, National Institutes of Health, Bethesda, MD 20892

[a] Current address: Department of Neuroscience, Perelman School of Medicine, University of Pennsylvania, Philadelphia, PA 19104

[b] Current address: Center for Cellular Immunotherapies, Perelman School of Medicine, University of Pennsylvania, Philadelphia, PA 19104

[#] These authors contributed equally to this work and should be considered as co-first authors. The order of appearance follows alphabetical order.

* Corresponding authors:

Xiaonan Zhao
Gene Structure and Disease Section
Laboratory of Cell and Molecular Biology
National Institute of Diabetes and Digestive and Kidney Diseases
National Institutes of Health, Bethesda, MD, USA
email: xiaonan.zhao@nih.gov

Karen Usdin
Gene Structure and Disease Section
Laboratory of Cell and Molecular Biology
National Institute of Diabetes and Digestive and Kidney Diseases
National Institutes of Health, Bethesda, MD, USA
email: karenu@nih.gov

Running title: The paradoxical effects of PMS2.

Keywords: microsatellite instability, MutLα, MutLγ, Huntington's disease, fragile X-related disorders

Supplementary Information

Supplemental Methods

RT-qPCR

Confluent mESCs were diluted 1:6, plated into 12-well tissue culture dishes and grown for 2 days with a medium change after 1 day. Total RNA was prepared using the RNeasy Mini Kit according to the manufacturer's directions (QIAGEN, Germantown, MD) and genomic DNA was removed by on-column digestion with DNAase I (New England Biolabs, Ipswich, MA). Reverse transcription was performed using SuperScript IV VILO Master Mix (ThermoFisher Scientific, Carlsbad, CA) as directed by the manufacturer. Quantitative PCR was performed using PowerUp SYBR Green Master Mix (ThermoFisher Scientific) with KiCqStart SYBR Green Primers (MilliporeSigma, Burlington, MA) targeting mouse *Pms2* (FM2_Pms2, 5'-GTTTCCGTTGACTCAGAATG-3' and RM2_Pms2, 5'-GCAGTATGCAGCTTTACTAAG-3') and targeting *Actb* as a reference gene (FM1_Actb, 5'-GATGTATGAAGGCTTTGGTC-3' and RM1_Actb, 5'-TGTGCACTTTTATTGGTCTC-3'). PCR conditions: 50°C for 2 minutes; 95°C for 2 minutes; 40 cycles of 95°C for 2 seconds, 57°C for 15 seconds, 72°C for 30 seconds; and followed by melt-curve analysis to assess the fidelity of the reactions.

Western blotting

Cells for western blotting were cultured in 6-well plates for 3 days with daily medium changes. Cells were rinsed with cold DPBS (Dulbecco's phosphate buffered saline), scraped, collected in cold DPBS with protease inhibitors, and pelleted. Cell pellets were lysed in RIPA buffer with

protease inhibitors and sonicated. For each sample, ~50 μg protein per lane was analyzed on 4-12% bis-tris gels in MOPS running buffer (ThermoFisher Scientific). Proteins were transferred to nitrocellulose membranes using Trans-Blot Turbo transfer buffer (Bio-Rad Laboratories, Hercules, CA). Antibodies were diluted in 5% ECL Prime Blocking Agent (Cytiva, Wilmington, DE) in tris-buffered saline: anti-FLAG M2 (1:1000; MilliporeSigma), goat anti-mouse IgG:DyLight 800 (1:2500, Bio-Rad Laboratories), hFAB Rhodamine Anti-Tubulin (1:10000, Bio-Rad Laboratories).

Allele Expansion Modeling

A time-course simulation was performed to model the repeat dynamics in a population of repeat-containing alleles. The initial population consisted of 1500 alleles each having the same number of repeats. The images shown incorporate the PCR stutter artifacts typical of repeat-containing sequences. At each simulated time point, every allele in the population has a defined probability of expanding, contracting, or remaining unchanged. Based on Møllersen et al. (1), the default probability of expansion was set to 0.026. The probability of a contraction occurring was set to be either zero or equal to the probability of expansion. Each expansion or contraction altered the repeat length by one unit. Allele populations were updated iteratively at each time point to reflect cumulative changes over time. The simulation was run for 301 iterations, at which point the modal repeat number in the population lacking contractions has increased by approximately six repeats, corresponding to the gain in repeats observed in the tail samples of

Mlh3 WT mice at 12 months of age (Figure S3C). The R script used for this simulation is available upon request.

Bulk RNA-seq analysis

Bulk RNA-seq gene expression data for hepatocytes were extracted from a study by Bragg et al. (PMC10488683) (2) obtained from the University of Washington's Library data repository (http://hdl.handle.net/1773/50042). The PMC10488683 dataset was normalized to transcripts per million (TPM). The expression levels of *Fmr1* and *Htt* in wild-type samples were compared. A paired two-tailed t-test was used to assess statistical differences in TPM-normalized expression between the two genes. Data analysis was performed in R, and all scripts used for processing and analysis are available at DOI: 10.5281/zenodo.15491084.

Single-cell RNA-seq analysis

Single-cell RNA-seq data from adult mouse testes were obtained from a published dataset (3). Cell types were classified into spermatogonia (SPGs), spermatocytes, and spermatids as defined in a previous study (3). Data processing and visualization were performed in R using the Seurat package (v5.2.1). Cells expressing either *Fmr1* or *Htt* were identified, and histograms were generated to show the distribution of expression across cells within each germ cell type. Proportions of cells expressing each gene were plotted with average expression levels indicated by vertical dotted lines. The code for this analysis is available at DOI: 10.5281/zenodo.15491084.

Supplemental figures and legends

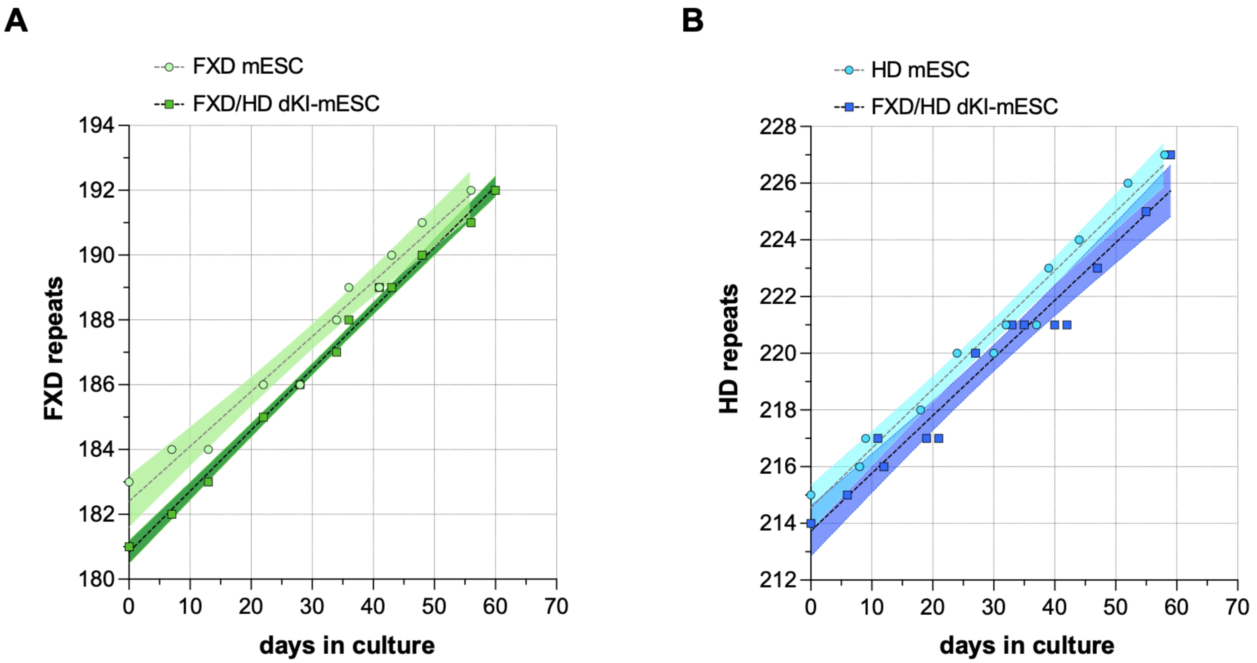

Cell line	Repeats/week	SD
FXD mESC	1.1	+/- 0.12
FXD/HD dKI-mESC	1.2	+/- 0.25
p value	0.51	

(Average of 3 FXD mESC lines and 6 FXD/HD double knock-in mESC lines.)

Cell line	Repeats/week	SD
HD mESC	1.7	+/- 0.39
FXD/HD dKI-mESC	1.9	+/- 0.43
p value	0.60	

(Average of 2 HD mESC lines and 6 FXD/HD double knock-in mESC lines.)

Figure S1. Repeat expansion of FXD and HD repeats in mESCs with either single or double repeat knock-in. (A) Expansion of FXD repeat across time in FXD mESCs and FXD/HD double knock-in (dKI) mESCs. (B) Expansion of HD repeat across time in HD mESCs and FXD/HD double knock-in mESCs. The area within the 95% confidence interval error bands were filled. Linear regression was performed with GraphPad Prism 10.2 to model the relationship between the repeat number changes and the days in culture, with the slope representing the rate of change of the repeats with respect to the day. The expansion rate per week was calculated by multiplying the expansion rate per day by 7. The repeat rate in single and double knock-in mESC

were compared using a two-tailed unpaired t-tests performed with GraphPad Prism 10.2. The adjusted *p*-value is listed in the table below.

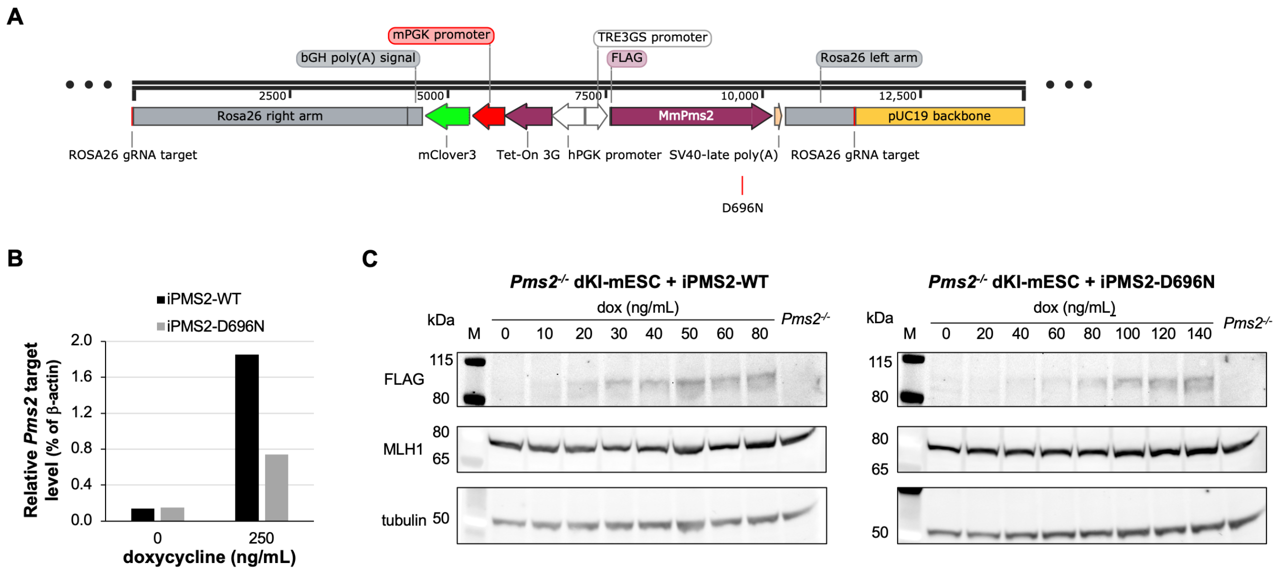

Figure S2. Doxycycline (DOX)-induced expression of FLAG-tagged *Pms2* constructs encoding either a WT (iPMS2-WT) or an endonuclease-deficient D696N mutant (iPMS2-D696N) form of PMS2. (A) Linear representation of the constructs used to generate iPms2-WT and -D696N lines. PGK promoters drive constitutive expression of doxycycline-responsive Tet-On 3G protein and a mClover3 fluorescent marker. In the opposite orientation, the TRE3GS promoter drives doxycycline-inducible expression of *Pms2*. The D696N mutation is indicated in red below the *Pms2* cDNA sequence. (B) Quantitative PCR of *Pms2* targets, expressed as a percentage of β-actin, from mESC lines carrying both constructs and treated with the indicated concentrations of doxycycline. (C) Western blots of whole-cell lysates from mESCs treated with the indicated concentrations of doxycycline and from *Pms2*$^{-/-}$ control mESCs. Blots were probed with antibodies indicated at left.

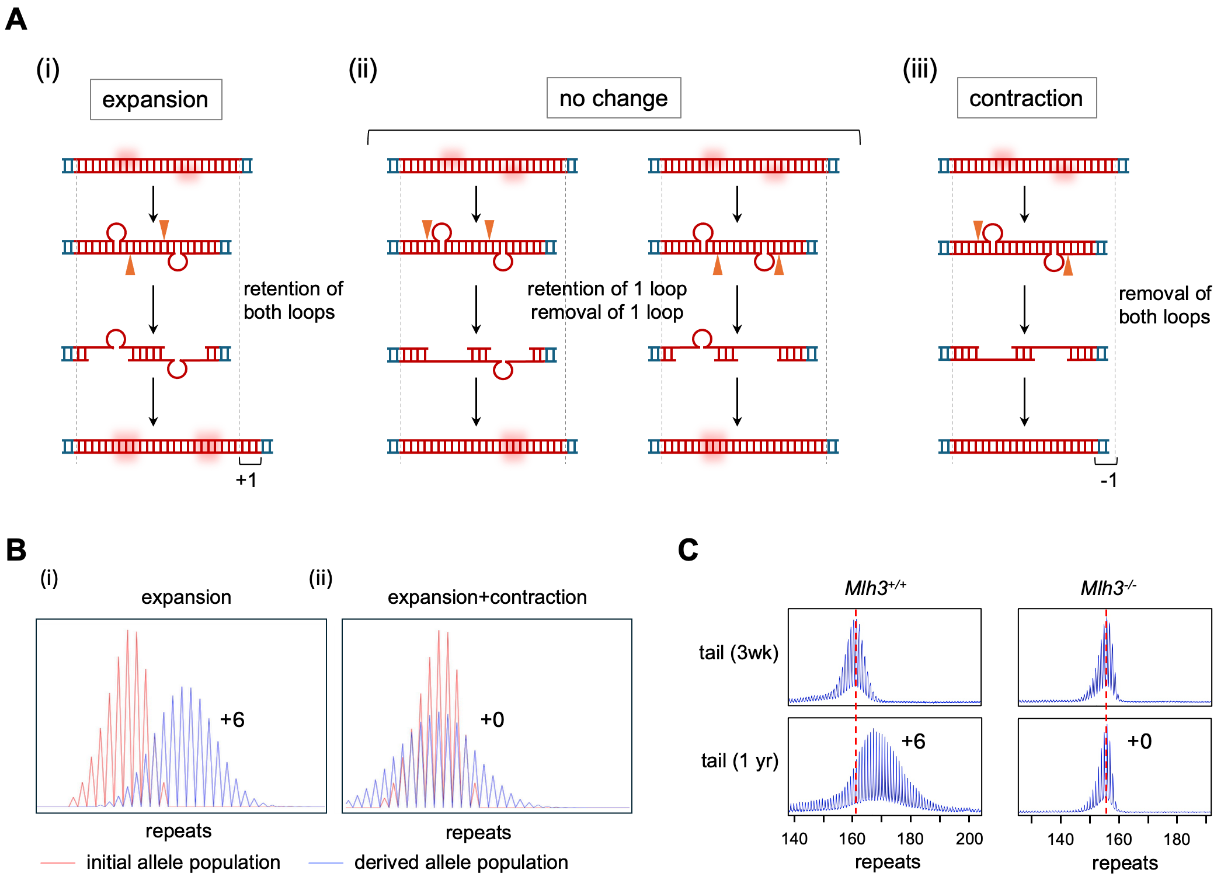

Figure S3. Independent loop-out processing by MutLα leads to predictions inconsistent with *in vivo* data. (A) Schematic representation of three possible outcomes when two loop-outs are processed independently by MutLα in the absence of MutLγ. The orange triangles represent MutLα cut sites. (i) When both cuts occur on the opposite strands of the loop-outs, similar to the MutLγ -mediated cleavage, gap-filling the two looped-out regions will result in expansion. (ii) When both cuts occur on the same strand, excision or strand-displacement results in the removal of one loop-out. After gap-filling by Polδ the original allele will be restored (no change). (iii) In the case that both cuts occur on the same strand of the loop-outs, both loop-outs will be removal and result in contraction. (B) Simulation of the change in the allele size distribution changes under two conditions. (i) A population undergoing expansions without contractions,

consistent with the coordinated model that expansion occurs only in the present of MutLγ. (ii) A population undergoing a combination of a similar number of expansions and contractions, modeling the scenario that in the absent of MLH3, MutLα process the substrates independently, as described in (A). In both simulations, the starting allele population was set to correspond to the profile seen in the tail DNA of Mlh3 null animals taken at weaning. The profiles shown represent the 301st iteration of the simulation, by which point the modal repeat number in the population lacking contractions (i) has increased by approximately 6 repeats, to allow comparison with the number of repeats added in the tail of a *Mlh3* WT mouse at 12 months of age shown in (C). Simulation details are described in Materials and Methods. (C) The PCR profiles of tail DNA from MLH3 WT and null mice at weaning and 12 months of age. The allele distribution in the MLH3 null mice does not match the simulation in (B-ii), suggesting that the independent processing model alone cannot fully account for the *in vivo* findings.

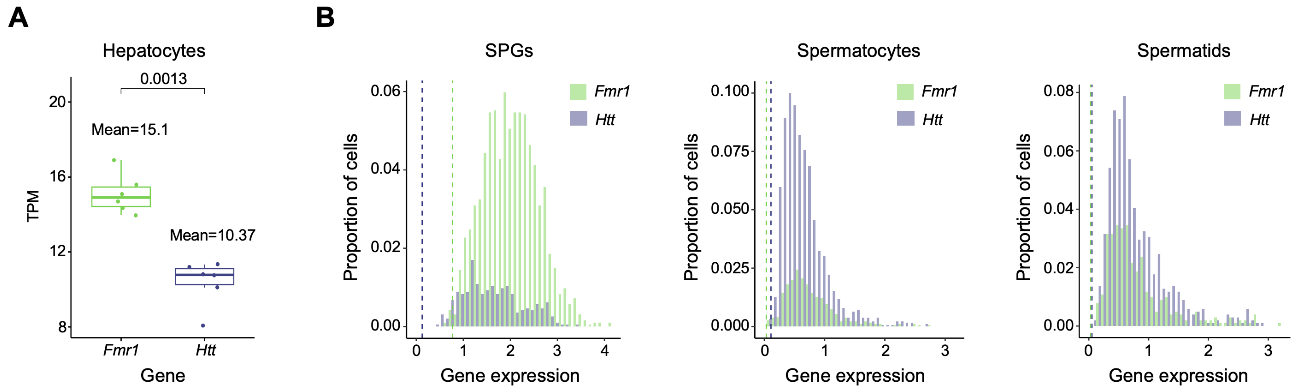

Figure S4. *Fmr1* and *Htt* expression in hepatocytes and spermatogenic cells. (A) Box plot comparing TPM-normalized expression of *Fmr1* and *Htt* in hepatocytes using bulk RNA-seq datasets. (B) Histograms showing the proportion of spermatogenic cells (SPGs, spermatocytes, and spermatids) expressing *Fmr1* (green) or *Htt* (blue), with dotted lines indicating the average expression level of each gene. See Materials and Methods for dataset sources and analysis details.

References

1. L. Mollersen, A. D. Rowe, E. Larsen, T. Rognes, A. Klungland, Continuous and periodic expansion of CAG repeats in Huntington's disease R6/1 mice. *PLoS Genet* **6**, e1001242 (2010).
2. R. M. Bragg *et al.*, Huntingtin loss in hepatocytes is associated with altered metabolism, adhesion, and liver zonation. *Life Sci Alliance* **6** (2023).
3. C. D. Green *et al.*, A Comprehensive Roadmap of Murine Spermatogenesis Defined by Single-Cell RNA-Seq. *Dev Cell* **46**, 651-667 e610 (2018).
