# Supplementary material for: PMS2 has both pro-mutagenic and anti-mutagenic effects on repeat instability in the Repeat Expansion Diseases
Source: bioRxiv. 2025 Apr 2:2024.08.13.607839. Originally published 2024 Aug 13. Preprint. [Version 2] doi: 10.1101/2024.08.13.607839 (PMC11343130; doi:10.1101/2024.08.13.607839)
Supplement: Supplementary file 1 [file media-1.pdf]

PMS2 has both pro-mutagenic and anti-mutagenic effects on repeat instability in the Repeat Expansion Diseases

Diego Antonio Jimenez[1,a,#], Carson J. Miller[1,#], Alexandra Walker[1,#], Kusala Anupindi[1,b], Karen Usdin[1,*], and Xiaonan Zhao[1,*]

[1] Section on Gene Structure and Disease, Laboratory of Cell and Molecular Biology, National Institute of Diabetes and Digestive and Kidney Diseases, National Institutes of Health, Bethesda, MD 20892
[a] Current address: Department of Neuroscience, Perelman School of Medicine, University of Pennsylvania, Philadelphia, PA 19104
[b] Current address: Center for Cellular Immunotherapies, Perelman School of Medicine, University of Pennsylvania, Philadelphia, PA 19104

# These authors contributed equally to this work and should be considered as co-first authors. The order of appearance follows alphabetical order.

* Corresponding authors:

Xiaonan Zhao
Gene Structure and Disease Section
Laboratory of Cell and Molecular Biology
National Institute of Diabetes and Digestive and Kidney Diseases
National Institutes of Health, Bethesda, MD, USA
email: xiaonan.zhao@nih.gov

Karen Usdin
Gene Structure and Disease Section
Laboratory of Cell and Molecular Biology
National Institute of Diabetes and Digestive and Kidney Diseases
National Institutes of Health, Bethesda, MD, USA
email: karenu@nih.gov

Running title: The paradoxical effects of PMS2.

Keywords: microsatellite instability, MutLα, MutLγ, Huntington's disease, fragile X-related disorders

Supplemental Methods

RT-qPCR

Confluent mESCs were diluted 1:6, plated into 12-well tissue culture dishes and grown for 2 days with a medium change after 1 day. Total RNA was prepared using RNeasy Mini Kit according to the manufacturer's directions (QIAGEN, Germantown, MD) and genomic DNA was removed by on-column digestion with DNAase I (New England Biolabs, Ipswich, MA). Reverse transcription was performed using SuperScript IV VILO Master Mix (ThermoFisher Scientific, Carlsbad, CA) as directed by the manufacturer. Quantitative PCR was performed using PowerUp SYBR Green Master Mix (ThermoFisher Scientific) with KiCqStart SYBR Green Primers (MilliporeSigma, Burlington, MA) targeting mouse *Pms2* (FM2_Pms2, 5'-GTTTCCGTTGACTCAGAATG-3' and RM2_Pms2, 5'-GCAGTATGCAGCTTTACTAAG-3') and targeting *Actb* as a reference gene (FM1_Actb, 5'-GATGTATGAAGGCTTTGGTC-3' and RM1_Actb, 5'-TGTGCACTTTTATTGGTCTC-3'). PCR conditions: 50°C for 2 minutes; 95°C for 2 minutes; 40 cycles of 95°C for 2 seconds, 57°C for 15 seconds, 72°C for 30 seconds; and followed by melt-curve analysis to assess the fidelity of the reactions.

Western blotting

Cells for western blotting were cultured in 6-well plates for 3 days with daily medium changes. Cells were rinsed with cold DPBS (Dulbecco's phosphate buffered saline), scraped, collected in cold DPBS with protease inhibitors, and pelleted. Cell pellets were lysed in RIPA buffer with protease inhibitors and sonicated. For each sample, ~50µg protein per lane was analyzed on 4-12% bis-tris gels in MOPS running buffer (ThermoFisher Scientific). Proteins were transferred to nitrocellulose membranes using Trans-Blot Turbo transfer buffer (Bio-Rad Laboratories, Hercules, CA). Antibodies were diluted in 5% ECL Prime Blocking Agent (Cytiva, Wilmington, DE) in tris-buffered saline: anti-FLAG M2 (1:1000; MilliporeSigma), goat anti-mouse IgG:DyLight 800 (1:2500, Bio-Rad Laboratories), hFAB Rhodamine Anti-Tubulin (1:10000, Bio-Rad Laboratories).

Supplemental figures

A

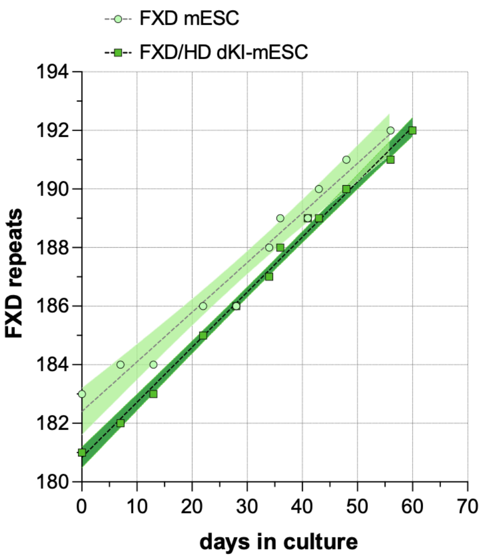

B

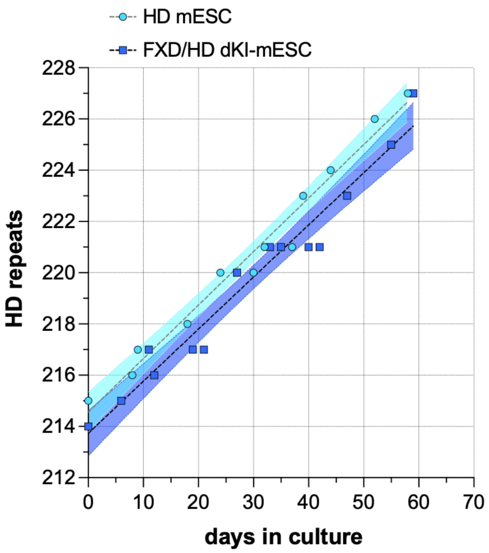

Cell line	Repeats/week	SD
FXD mESC	1.1	+/- 0.12
FXD/HD dKI-mESC	1.2	+/- 0.25
p value	0.51	

(Average of 3 FXD mESC lines and 6 FXD/HD double knock-in mESC lines.)

Cell line	Repeats/week	SD
HD mESC	1.7	+/- 0.39
FXD/HD dKI-mESC	1.9	+/- 0.43
p value	0.60	

(Average of 2 HD mESC lines and 6 FXD/HD double knock-in mESC lines.)

Fig. S1. Repeat expansion of FXD and HD repeats in mESCs with either single or double repeat knock-in. (A) Expansion of FXD repeat across time in FXD mESCs and FXD/HD double knock-in (dKI) mESCs. (B) Expansion of HD repeat across time in HD mESCs and FXD/HD double knock-in mESCs. The area within the 95% confidence interval error bands were filled. Linear regression was performed with GraphPad Prism 10.2 to model the relationship between the repeat number changes and the days in culture, with the slope representing the rate of change of the repeats with respect to the day. The expansion rate per week was calculated by multiplying the expansion rate per day by 7. The repeat rate in single and double knock-in mESC were compared using a two-tailed unpaired t tests performed with GraphPad Prism 10.2. The adjusted *p*-value is listed in the table below.

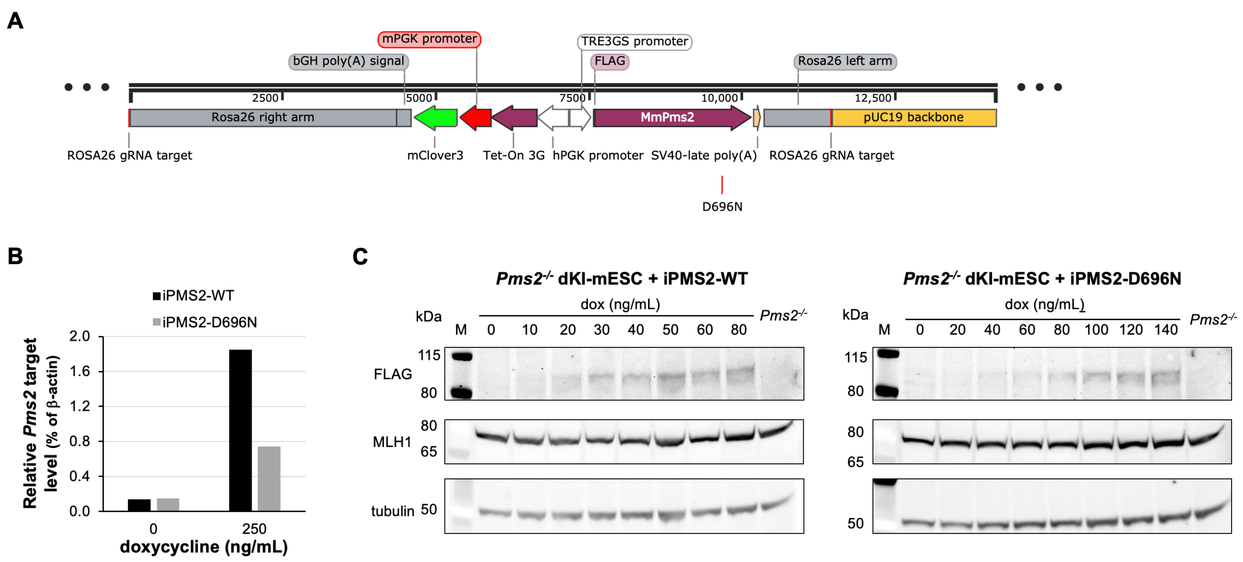

Fig. S2. Doxycycline-induced expression of FLAG-tagged *Pms2* constructs, encoding either a WT (iPMS2-WT) or an endonuclease-deficient D696N mutant (iPMS2-D696N) form of PMS2. (A) Linear representation of the constructs used to generate iPms2-WT and -D696N lines. PGK promoters drive constitutive expression of doxycycline-responsive Tet-On 3G protein and a mClover3 fluorescent marker. In the opposite orientation, the TRE3GS promoter drives doxycycline-inducible expression of *Pms2*. The D696N mutation is indicated in red below the *Pms2* cDNA sequence. (B) Quantitative PCR of *Pms2* targets, expressed as a percentage of b-actin, from mESC lines carrying both constructs and treated with the indicated concentrations of doxycycline. (C) Western blots of whole-cell lysates from mESCs treated with the indicated concentrations of doxycycline and from *Pms2*$^{-/-}$ control mESCs. Blots were probed with antibodies indicated at left.
