# Supplementary material for: PMS2 has both pro-mutagenic and anti-mutagenic effects on repeat instability in the Repeat Expansion Diseases
Source: bioRxiv. 2024 Aug 13:2024.08.13.607839. Preprint. [Version 1] doi: 10.1101/2024.08.13.607839 (PMC11343130; doi:10.1101/2024.08.13.607839)
Supplement: Supplementary file 1 [file media-1.pdf]

PMS2 has both pro-mutagenic and anti-mutagenic effects on repeat instability in the Repeat Expansion Diseases

Alexandra Walker[1,#], Diego Antonio Jimenez[1,a,#], Karen Usdin[1,*], and Xiaonan Zhao[1,*]

[1] Section on Gene Structure and Disease, Laboratory of Cell and Molecular Biology, National Institute of Diabetes and Digestive and Kidney Diseases, National Institutes of Health, Bethesda, MD 20892

[a] Current address: Department of Neuroscience, Perelman School of Medicine, University of Pennsylvania, Philadelphia, PA 19104

# These authors contributed equally to this work and thus should be considered co-first authors.

* Corresponding authors:

Xiaonan Zhao
Gene Structure and Disease Section
Laboratory of Cell and Molecular Biology
National Institute of Diabetes and Digestive and Kidney Diseases
National Institutes of Health, Bethesda, MD, USA
email: xiaonan.zhao@nih.gov

Karen Usdin
Gene Structure and Disease Section
Laboratory of Cell and Molecular Biology
National Institute of Diabetes and Digestive and Kidney Diseases
National Institutes of Health, Bethesda, MD, USA
email: karenu@nih.gov

Running title: The paradoxical effects of PMS2.

Keywords: microsatellite instability, mismatch repair (MMR), MutLα, MutLγ, Huntington's disease, Fragile X-related disorders.

Supplementary Figure

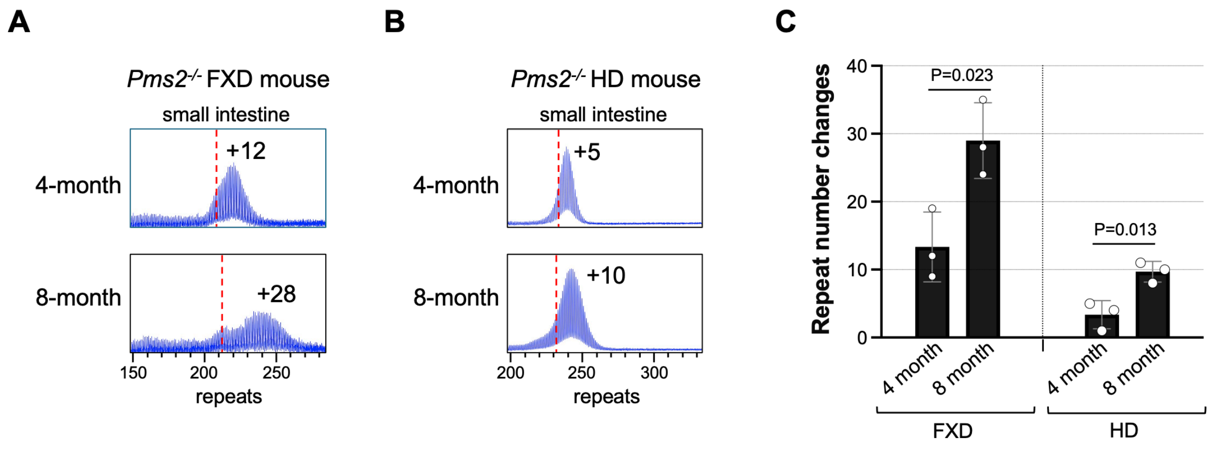

Fig. S1. Expansion accumulates with age in small intestine of Pms2 null animals. (A) Representative repeat PCR profiles of small intestine and colon from 4- and 8-month-old *Pms2*$^{-/-}$ FXD male mice with ~210 repeats. (B) Representative repeat PCR profiles of small intestine from 4- and 8-month-old *Pms2*$^{-/-}$ HD male mice with ~230 repeats. The number associated with each profile indicates the change in repeat number relative to the original inherited allele. The dashed lines represent the sizes of the original inherited alleles as ascertained from the tail DNA taken at 3 weeks. (C) Comparison of the repeat number changes in the small intestine of 4- and 8-month-old *Pms2*$^{-/-}$ mice. The 4-month-old FXD mice data represents the average of 3 *Pms2*$^{-/-}$ mice with 199-238 repeats (average of 214 repeats) in the original allele. The 8-month-old FXD mice data represents the average of 3 *Pms2*$^{-/-}$ mice with 204-212 repeats (average of 208 repeats) in the original allele. The 4-month-old HD mice data represents the average of 3 *Pms2*$^{-/-}$ mice with 227-234 repeats (average of 230 repeats) in the original allele. The 8-month-old FXD mice data represents the average of 3 *Pms2*$^{-/-}$ mice with 229-234 repeats (average of 232 repeats) in the original allele. The error bars indicate the standard deviations of the mean. Each dot represents one animal. In each mouse model, the repeat number changes at different age were compared using a t test performed with GraphPad Prism 10.2. P-values are listed in the graph.
